# Technical Functions of Digital Wearable Products (DWPs) in the Consumer Acceptance Model: A Systematic Review and Bibliometric Analysis with a Biomimetic Perspective

**DOI:** 10.3390/biomimetics10080483

**Published:** 2025-07-22

**Authors:** Liu Yuxin, Sarah Abdulkareem Salih, Nazlina Shaari

**Affiliations:** Faculty of Design and Architecture, Universiti Putra Malaysia (UPM), Serdang 43400, Malaysia

**Keywords:** bio-inspired wearables, biomimetic principles, consumer behavior, digital wearable products, medical wearable robotics, technology acceptance model, wearable technology

## Abstract

Design and use of wearable technology have grown exponentially, particularly in consumer products and service sectors, e.g., healthcare. However, there is a lack of a comprehensive understanding of wearable technology in consumer acceptance. This systematic review utilized a PRISMA on peer-reviewed articles published between 2014 and 2024 and collected on WoS, Scopus, and ScienceDirect. A total of 38 full-text articles were systematically reviewed and analyzed using bibliometric, thematic, and descriptive analysis to understand the technical functions of digital wearable products (DWPs) in consumer acceptance. The findings revealed four key functions: (i) wearable technology, (ii) appearance and design, (iii) biomimetic innovation, and (iv) security and privacy, found in eight types of DWPs, among them smartwatches, medical robotics, fitness devices, and wearable fashions, significantly predicted the customers’ acceptance moderated by the behavioral factors. The review also identified five key outcomes: health and fitness, enjoyment, social value, biomimicry, and market growth. The review proposed a comprehensive acceptance model that combines biomimetic principles and AI-driven features into the technical functions of the technical function model (TAM) while addressing security and privacy concerns. This approach contributes to the extended definition of TAM in wearable technology, offering new pathways for biomimetic research in smart devices and robotics.

## 1. Introduction

The rapid development of the Internet of Things (IoT) has led to the emergence of compact electronic and computing devices and robotics that can be embedded in or worn on the human body, known as smart wearable devices or wearable technology [1]. These technologies integrate intelligent computing devices into accessories like clothing and fashion items, aiming for seamless daily integration [2]. Fitness trackers and smartwatches are considered the next ubiquitous technology following the smartphone [3]. Smart wearables are prevalent in daily life computing in lifestyle, healthcare, sports, and personal safety. They enable the management of personal information through their ability to monitor, store, and transmit individual-related health and physical activity information, such as body temperature, blood pressure, heart rate, caloric intake and expenditure, step count, sleep patterns, and location [4]. As digital technology rapidly evolves, these devices signify a substantial shift in consumer interaction with technology [4,5,6,7]. Recently, wearable robotics and intelligent assistive technologies have been quickly introduced to healthcare sectors and data biomechanics, driven by the convergence of biomedical engineering, artificial intelligence (AI), and sensor technology [8,9,10,11]. These systems were primarily designed to support human physical abilities and to enhance the performance and safety of users in occupational settings [8].

The global digital wearable device market has continuously grown and is forecasted to reach USD 186.14 billion by 2030 with a CAGR of 14.6% from 2023 to 2030 [5]. The progress in miniaturization, sensors, data analytics, and AI and the increasing awareness and involvement of consumers in health care, sports, and enterprise contexts also drive the enlargement [5]. Smart wearables, such as smartwatches and smart rings, have gained significant market attention. IDC [6] predicts that global shipments will increase from 506.5 million units in 2023 to 559.7 million units by 2024, of which wearable wristbands will increase from 18.5 million units to 19.8 million units [5]. By 2024, the global wearable market is expected to reach $109 billion, with a CAGR of 15.1% from 2021 to 2024 [7], with the Asia-Pacific region holding the largest share by 2026 [8]. China was the largest market for smart wearables, with the sports wearables market reaching $4.7 billion in 2023 [9].

The intersection of technological progress and evolving consumer expectations has transformed the marketing and positioning of wearable technology. Nieroda et al. [10] emphasized that the integration of digital intelligence assistance with conventional consumer products has resulted in the creation of multifunctional hybrids, necessitating innovative design and technology. This transition transcends technical sophistication; consumer adoption depends on the efficacy of the digital wearable products (DWPs) in aligning with user wants, aspirations, and societal norms [11,12,13,14,15]. Technological advancements like AI integration and biomimetic innovation have contributed to enhancing functionality; yet, perceived ease of use (PEOU), privacy concerns, and social acceptability are essential elements influencing customer acceptance [4,16,17,18,19,20]. Biomimetic innovation in wearable technology refers to the application of biological principles, forms, and systems to enhance the design, functionality, and user experience of devices. This concept encompasses features inspired by nature, such as self-healing materials, biomimetic skin structures, and designs that draw inspiration from biological metabolism or natural processes [21,22,23,24,25]. Thus, this concept is grounded in the field of biomimetics, which studies how biological systems and organisms can inspire engineering and design solutions to improve products [24,25].

Recent literature has increasingly emphasized the adoption of wearable technologies, with scholarly research in this domain growing to examine wearable technology [4]. Niknejad et al. [11] offered a thorough examination of the smart wearable sector, emphasizing the substantial growth in research. However, they recommended the necessity for further investigation to fill existing gaps and limitations, as well as to enhance comprehension of the broader implications of these technologies across different sectors. Ferreira et al. [12] analyzed the literature on wearable technology to identify trends and themes, highlighting the interdisciplinary character of wearable science. Yet, they highlighted the lack of integration between the DWPs and consumers’ perspectives of this research field. Chakraborty et al. [13] studied the application of wrist-worn wearable devices in disease management. Keshet et al. [14] underscored the potential of wearable and digital devices in the monitoring and control of metabolic diseases. They contend that these technologies provide ongoing and longitudinal health monitoring beyond clinical environments, which is essential for early identification and prompt management. However, Chakraborty et al. [13] and Keshet et al. [14] only focused on the healthcare and medical discipline of the DWPs. Kalantari [4] examined the prospective advantages of wearable technologies in healthcare and assistive services, indicating that although these technologies could transform societies and industries, their adoption rate has not aligned with predictions. Ometov et al. [8] highlighted the significant issues that have emerged in wearable technology, namely those related to data processing, privacy, and security. Resolving these difficulties was crucial for the effective incorporation of wearable technologies into everyday life, facilitating greater acceptance and use [8]. Both studies focused only on one dimension of the technical function of DWPs (either perceived usefulness [4] or privacy and security [8]).

Despite the increasing interest in wearable technology, the existing studies on the typology of DWPs remain fragmented, with a specific focus often limited to particular (single) disciplines (e.g., medical or fitness) [13,14,26,27,28] or form or function factors (e.g., watches or textiles) [11,29,30,31,32,33,34]. The current typology of wearable technology focuses only on identifying the types, their trends, and performance and lacks a comprehensive understanding of the technical functions in consumer acceptance [28,35]. Besides, there is a lack of studies on how different emerging dimensions of technical functions, including biomimetic innovation, affect consumer acceptance decisions in the wearable products market [24,28,35,36]. The current literature has either examined the technical functions of DWPs [4,8,15] or the variables influencing customer acceptance separately [4]. For example, advanced biomimetic features in wearable devices may fail if users perceive them as too complex or difficult to use [24,25,28]. Without integrating both technological innovation and user acceptance perspectives, even technically superior devices risk market rejection and underutilization. At the same time, several recent systematic reviews have concentrated on specific health-related applications (health devices in health care) [4,8,11]. This fragmented approach limits our understanding of how technological innovation and user perceptions interact. The comprehensive understanding of the different types of DWPs and their emergent technical function in the acceptance model is essential because it enables researchers to improve the consumer acceptance models for emerging technologies while helping designers reduce adoption barriers and deliver products that better meet consumer needs.

The existing studies have widely applied the technology acceptance model (TAM) but have not incorporated emerging technologies such as biomimetic innovation into the TAM [37]; this might limit the comprehensive understanding and application of the acceptance model in the emerging technology. Biomimetic principles, particularly biomimetic technological innovation (BTI), are still underexplored in the context of DWPs adoption models. With advancements in bio-inspired sensors, bio-inspired structures, and adaptive interfaces, integrating biomimicry principles into the design of DWPs could enhance the understanding of the TAM and enhance long-term sustainability while improving functionality and user experience [11,26,27]. Besides, although individual studies identify the outcomes of DWP use [26,38,39,40], there is a lack of an inclusive framework that synthesizes the DWP perceived outcomes based on their technical functions in the acceptance model, which hinders the development of comprehensive, integrated acceptance models [38,39]. Therefore, there is a need for a thorough understanding of the technical functions of different types of DWPs and their perceived outcomes in the consumer acceptance model.

Therefore, this study made significant contributions to this knowledge gap by understanding the comprehensive and identifying various technical functions of different types of DWPs in the consumer acceptance model and identifying their perceived outcomes. A comprehensive systematic literature review with a bibliometric, descriptive, and thematic analysis using a rigid systematic approach [40,41,42,43,44] was conducted on 38 full-text journal articles published in peer-reviewed journals in English over the past 10 years to answer the following questions: What are the different types of DWPs and their related emergent technical functions in the consumer acceptance model? How do these emergent technical functions influence consumers’ acceptance and behavior of DWPs? What are the related outcomes of DWPs and their emergent technical functions in the consumer acceptance model? By answering this, the current SLR contributes to filling the gap in the existing literature and clarifying the fragmented landscape by providing a comprehensive understanding of the typology of DWPs, linking their technical functions and related outcomes to the consumer acceptance model. Understanding this interaction is essential for explaining how innovations influence user perceptions, attitudes, and acceptance; in turn, this helps researchers design user-friendly features and facilitates industry and companies to accelerate responsible adoption. The current review also contributes to the existing reviews by explicitly integrating biomimetic innovation into the consumer acceptance model. Therefore, it contributes significantly to linking the wearable technology industry to consumer acceptance and fostering sustainable growth in the robotics market.

The following sections are included: Section 2, the “Literature Review” discusses the existing studies related to technical functions in DWPs, the concept of BTI, and the theoretical basis of the study. Section 3 includes the “Materials and Methods,” providing details of the SLR methodology used, PROSPERO registration, search strategy, inclusion and exclusion criteria, data extraction and synthesis, and quality appraisal of the selected studies. Section 4 involves the “Results” of the study, providing a detailed report of the main results regarding the characteristics of the selected articles, co-authorship, keyword co-occurrence network results, DWPs types related to the acceptance model, technical functions of DWPs, consumer acceptance and related factors, and perceived outcomes of the DWPs in the consumer model. Finally, Section 5, “Discussion,” provides a detailed discussion of the key findings to answer the review questions. It also discusses the limitations, recommendations for future studies, conclusion, and contributions of this systematic review.

## 2. Literature Review

### 2.1. Technical Functions in DWPs

Wearable technology or wearable devices, also called digital wearables, are an electronic device type that is worn on or adjacent to the human body, collecting, processing, and transmitting information continuously [8]. Wearable devices can be characterized by their ability to seamlessly integrate into users’ daily lives, typically providing real-time feedback and data [16]. The term DWPs refers to a diverse array of items, including but not limited to smartwatches, fitness trackers, smart glasses, head-mounted displays, smart clothing, e-textiles, health monitoring devices, and smart jewelry [45,46,47,48,49]. Digital wearables have evolved into far more than compact electronic accessories; they now function as responsive, intelligent systems capable of mediating between individuals and their immediate environments. Meanwhile, wearable robotics refers to more specific wearable devices engineered to mimic the form and function of human limbs, providing additional forces to surpass human physical limitations. Early investigations identified their core characteristics, mobility, persistent connectivity, and contextual responsiveness, but the scope of what these devices can achieve has expanded significantly [4,16,50,51,52,53]. The uniqueness of these devices lies in their clever integration of sensors, compact processors, and wireless modules that together collect and process data related to the user’s physiological state, behavior, and environmental exposure [15]. Interestingly, it is not just the hardware that has evolved. Recent progress in miniaturization, power-efficient design, and big data analytics has fundamentally redefined what these tools can offer [11,54,55,56,57]. The trajectory has shifted from rudimentary tools, pedometers being an early example, to multifaceted platforms that incorporate AI-driven capabilities, including health tracking, biometric authentication, and augmented reality features, all of which operate within a broader connected technology ecosystem [8,58,59].

The technical functions of DWPs played a critical role in shaping both adoption and sustained use. Of particular relevance is their capacity to process time-sensitive data inputs and translate them into actionable feedback [15]. Rather than merely aggregating metrics, contemporary devices, such as smartwatches, fitness trackers, and medical sensors [60,61,62], interpret biometric data such as heart rate variability, circadian fluctuations, and kinetic activity, often converting these into tailored recommendations aimed at promoting behavioral change [17,62,63]. The integration of machine learning (ML) and intelligent assistive models into wearables is a significant advancement, allowing them to detect patterns and predict risk conditions before clinical manifestation [18,34,54]. While these models are not infallible, they have demonstrated value in contexts such as sleep disruption analysis and early cardiovascular anomaly detection. Additionally, deep learning computational models have contributed to greater accuracy in real-time signal classification, thereby enhancing the diagnostic and preventive potential of wearable systems across medical and consumer health contexts [19,30,34,46].

Another crucial function of DWPs is their seamless integration within IoT ecosystems. Contemporary wearables were engineered to interact with other smart devices, permitting the creation of harmonized data exchanges across multiple platforms [20]. For instance, smart glasses, bright rings, and integrating service robots communicate with home automation hubs, cloud databases, and smartphones to enable users to access a more interconnected digital environment [21,34,35,36,38,39,40,41,42,43,44,45,46,47,50,51,52,53]. The emergence of 5G networks has further bolstered connectivity, facilitating the transmission of high-frequency data in real time with minimal latency [22]. This extent of integration enhances user convenience, reinforcing the perception of wearables as essential instruments for everyday living.

Furthermore, recent progress in bio-integrated and energy-efficient wearables has broadened the prospective applications of wearable technology. Researchers have investigated the employment of flexible, biocompatible materials that permit sensors to adapt to the human body with limited discomfort [23,49]. Developments in energy harvesting (in other words, self-sustaining sensors powered by body motion or temperature gradients) have also improved the convenience and sustainability of wearables [24,35]. These developments indicate the increasing significance of wearables not solely in personal health monitoring but also medical diagnosis and rehabilitation [25,39].

Both the overall in-use experience and the functional performance of the product have influenced user attitudes and behaviors. Pancar and Ozkan [26] argued that attitude toward use is a key predictor of actual usage behavior. Erzetic et al. [27] noted that the simplicity of navigation, combined with efficient and accurate real-time monitoring, significantly enhances user engagement. Their simplicity has helped fuel increasing rates of usage, especially in fitness and health uses. As digital wearables continue to evolve, their success will largely depend on striking the right balance between advanced technical functions, usability, and consumer-centric design.

Despite rapid advancements and increased research in the technical capabilities of digital wearables, including real-time data analytics, AI integration, IoT connectivity, and energy-efficient designs, there remains a gap in understanding how these technical functions influence user engagement and consumer acceptance from a comprehensive perspective. While usability is often cited as a key factor, the extent to which advanced technical features directly contribute to user acceptance value has not been comprehensively studied. Therefore, this study will examine the influence of technical functions on user acceptance of DWPs.

### 2.2. Biomimetic Technological Innovation (BTI)

Biomimetic innovation is the development of novel technologies, materials, systems, or designs that intentionally emulate biological principles, structures, or functions, aiming to benefit humans, minimize environmental impacts, and facilitate a more harmonious relationship between humans and nature [24,25,28]. In biomimetic strategies, innovation is explicitly derived from and justified by a natural model, as evidenced by direct references to biological strategy in its design, function, or optimization. Thus, the key indicators of biomimetic innovations include self-healing mechanisms, skin structural mimicry, thermal regulation systems, and biomimetic design structures (e.g., nature-inspired design forms, animal muscles, animal skin adhesion, plant mimicking surface structures, and thermal regulation mimicking from plants and animal skin) [25,28,36].

Recent innovations in digital wearables have increasingly incorporated biomimetic technological advancements, drawing inspiration from biological systems to refine device functionality, efficiency, and user experience [25,28]. For instance, biomimetic technologies within wearable electronics focus on improving sensor sensitivity, material flexibility, and power efficiency, responding to the shifting demands of consumers for more seamless integration and sustainability [29]. A significant use of biomimetic technology in wearables was the creation of sensors that emulate biosensors, utilizing the human skin’s sensory capabilities with sophisticated touch feedback, body motion detection, and physiological monitoring [30]. Artificial electronic skins (e-skins), designed to emulate biological skin, integrate flexible and elastic sensors that provide real-time health monitoring while maintaining comfort [25,31]. These advancements have enhanced the precision of wearables in detecting biometric signals, hence giving significant advantages for healthcare and the enhancement of sports performance, among others.

Biomimetic approaches have also resulted in self-sustaining wearable devices and robotics that employ bio-inspired energy harvesting techniques, including triboelectric nanogenerators (TENGs) and piezoelectric materials, which generate energy from bodily movements [32]. These technologies reduce the necessity for traditional batteries, hence improving durability and fostering sustainability. Biomimetic energy solutions match with global sustainability objectives by decreasing the necessity for frequent battery changes and mitigating electronic waste [33,35]. Furthermore, biomimetic design concepts have enhanced adaptive and intelligent wearables by integrating AI-driven pattern recognition systems inspired by neural networks and biological cognition. Wearables employed these technologies to provide feedback while considering particular environmental or physiological situations by modifying their biometric monitoring capabilities [33]. On the other hand, the development of bionic wearable robotics in healthcare represents significant progress in intelligent assistive technology, seeking to better human health and mobility through biologically inspired design and artificial actuation. Drawing from biological models, these wearables included bio-inspired mechanical structures, adaptive control systems, and skin-mimicking structures to enable seamless human-robot interaction [16,21].

The sustainability biomimetic framework of DWPs might encompass energy-efficient functionality and environmentally friendly components that comply with circular economic principles. Ramasubramanian et al. [34] investigated biodegradable and recyclable materials in the creation of wearables, thereby mitigating the environmental consequences of gadget disposal. Gurova et al. [35] proposed that biomimetic prolonging product lifespan might enhance the modularity and reparability of wearables, sustainability, and diminish electronic waste.

Despite the considerable advancements in technical functions and sustainability potential of digital wearables and robotics through biomimetic technologies, including innovations in sensory accuracy, self-powered systems, and biodegradable materials, there exists a paucity of research assessing the influence of these biomimetic attributes on user perceptions of sustainability. Incorporating biomimicry in technological innovation and DWPs is a nascent subject requiring additional exploration, particularly with the consumers’ acceptance model. Therefore, this study will investigate how biomimetic features affect the users’ acceptance of DWPs.

### 2.3. Theoretical Basis of the Study

**Figure 1:**
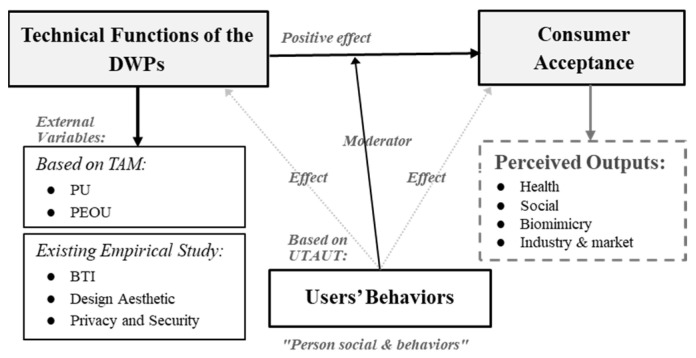
Conceptual framework for consumer acceptance of DWPs, based on consumer acceptance model [8,83].

The theoretical foundation of this study was established through an extensive review and analysis of previous studies (with the aid of bibliometric analysis using VOSviewer software, version 1.6.20) and under the framework of the existing theories of digital products and consumer acceptance models. The theoretical framework illustrated in Figure 1 is based on previous models of technology adoption and augmented by a biomimetic innovation perspective. The framework predominantly relied on the TAM [36] and the integration of BTI articulated by Benyus [36] with the integration of the previous study results [1,9,38,39].

TAM, introduced by Davis (1989) [36], remains one of the most influential frameworks for understanding user acceptance of information systems and emerging technologies. Its core constructs, perceived usefulness (PU) and PEOU, provide a robust basis for predicting user attitudes, behavioral intention, and actual system acceptance. TAM’s strengths lay in its simplicity and empirical validity across various technological contexts, including e-health applications, smart devices, and digital wearables [9,38,39].

In the context of advancing technologies such as biomimetic wearable devices, the TAM model might necessitate contextual modification to encompass emergent structures; this includes the incorporation of developing concepts such as perceived sustainability and biomimetic value (BTI), which signify people’s increasing fascination with ecological design and nature-inspired innovation [36]. BTI, as articulated by Janine Benyus in Biomimicry: Innovation Inspired by Nature (1997) [36], presents a paradigm shift in design and technology development by advocating for solutions modeled on natural processes, systems, and elements. BTI was progressively integrated into contemporary DWPs, showcasing advancements such as self-sustaining sensors, adaptive materials, and energy-efficient systems [1,22,28]. BTI highlighted technological components and materials inspired by biological systems that aim to improve performance, ergonomics, and sustainability of the products [22,28,31], which might influence consumer behavior and acceptance [31,64,65,66,67,68,69]. Therefore, technological innovation in biomimicry, also known as “biomimetic innovation,” refers to advanced technologies, materials, or systems inspired by biological principles or functions. Its distinguishing indicators include functional designs based on biological forms, self-healing capabilities, structural adaptations that mimic biological skins and surfaces, and thermal regulation systems inspired by animals or plants [24,25,28].

Moreover, additional factors have increasingly affected technological acceptance, such as design and perceived aesthetics [33,70,71,72,73], privacy, and data security issues [34,74,75,76]. Technological innovation in biomimicry, including eco-innovative design, has been shown to improve user engagement and acceptability of sustainable technology products [23,28,31,32]. These enhancements not only augment the explanatory capacity of TAM but also connect the model more closely with the intricate decision-making processes of contemporary users engaging with biologically inspired intelligent technologies. Privacy and data security markedly improved user acceptance and confidence in DWPs. Given that these devices frequently gather sensitive personal information, such as health indicators, location, and behavioral patterns, users are becoming progressively apprehensive about storing, sharing, and safeguarding their data [8,77,78]. Consumers’ readiness to adopt and persist in using DWPs escalate when they recognize the presence of strong privacy protections and security protocols [33,79]. Privacy assurance is recognized as a crucial factor in perceived trustworthiness, which subsequently enhances technological acceptability [8,74,75,80].

The perceived aesthetics of product design play a critical role as a technological factor influencing consumer purchase intention and the acceptance of DWPs. As visual design features increasingly influenced customer emotional, enjoyment, and cognitive responses, aesthetically appealing products were more likely to be regarded as innovative, trustworthy, and user-friendly, enhancing their marketability and acceptance [23,27,33,75,76,81]. Aesthetic appeal and well-designed products enhance first perceptions and bolster perceived quality and personal connection with the product, especially in wearable technology where fashion and functionality converge [27,33,71]. The biomimetic principles might also shape design aesthetics. For example, forms modeled after organic geometries or forms inspired by nature can contribute to shaping users’ internal product standards and affect emotional and symbolic value perception [24,28,31,32,36,81].

On the other hand, consumer behavior and social influence also offer a robust explanation for the acceptance of digital products, including DWPs [12]. Ultimately, behavioral attitude leads to product acceptance and purchase intention. A variety of technology acceptance theories, such as the unified theory of acceptance and use of technology (UTAUT) [39], emphasize the role of users’ behavior and social influence, the degree to which individuals perceive that essential others believe that they should use the technology, as a significant predictor of behavioral intention. This variable is especially relevant in socially visible technologies like wearables, where peer influence, trends, and normative beliefs shape user adoption decisions [39]. More specifically, users’ or consumers’ behavioral attitude, a user’s personal preference, and evaluation of a technology are crucial mediators between external variables (such as PU, data security, and design aesthetics) and digital products acceptance. When users perceive a product to be beneficial, socially endorsed, and well-designed, they are more likely to form favorable attitudes, ultimately leading to higher acceptance and purchase behavior [39].

Therefore, incorporating design aesthetics, data security, biomimetic technology innovation (BTI), and users’ behavioral attitude into the TAM offered a more comprehensive and contextual framework for understanding technology acceptance behavior for DWPs [37,39,82,83,84,85,86,87,88]. These added factors addressed the evolving expectations of modern consumers, particularly in the realm of wearable technologies. Figure 1 shows the conceptual model of this study, in which DWPs’ technical function characteristics, including PU, PEOU, design aesthetics, safety and security, and BTI (or biomimetic innovation), play a critical role as external variables affecting the consumer’s acceptance of the DWPs, moderated by the consumer behavior, leading to related outcomes. This integrated approach allows for a more nuanced exploration of user adoption dynamics, particularly in the context of emerging, eco-innovative, and visually expressive wearable technologies.

## 3. Materials and Methods

This review employed a rigorous systematic approach to ensure a comprehensive and unbiased analysis of the literature, following a structured review protocol and focusing on empirical evidence. The systematic review and bibliometric, thematic, and descriptive analyses (with the aid of tabular and figural analyses) were conducted according to the PRISMA guidelines by Moher et al. [40]. The review addressed the following research questions: hat are the different types of DWPs and their related emergent technical functions in the consumer acceptance model? How do these emergent technical functions influence consumers’ acceptance and behavior of DWPs? What are the related outcomes of DWPs and their emergent technical functions in the consumer acceptance model?

**Figure 2:**
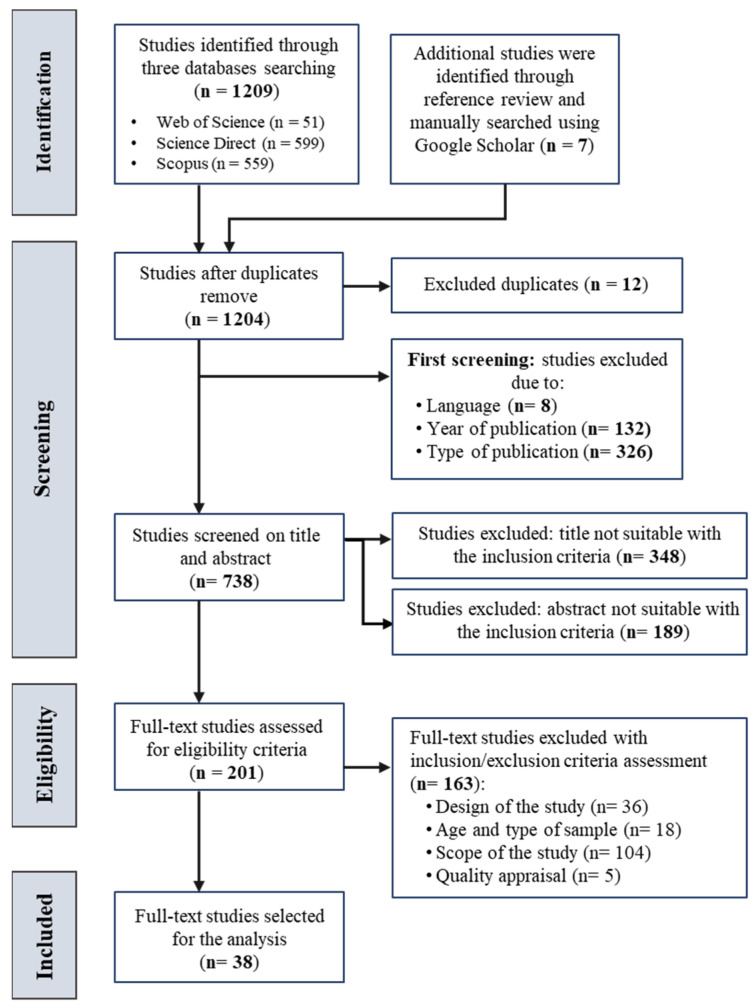
PRISMA flowchart for the selected articles.

PROSPERO was searched to confirm that no similar systematic review protocol had been registered, ensuring the uniqueness of this study. The systematic review protocol has been registered and accepted in PROSPERO (ID: CRD420251070908; available at https://www.crd.york.ac.uk/PROSPERO/view/CRD420251070908, accessed on 11 July 2025). A PRISMA framework was used for data identification, screening, and eligibility, as illustrated in Figure 2 and detailed in Supplementary Information, Table S3. Bibliometric analysis was combined to identify the key themes (topics) of the review to validate the key themes used in the thematic analysis of this review study. The primary data analysis and synthesis of the selected studies were carried out using thematic analysis (including theme, category, and code), descriptive analysis (frequency and percentage), and tabular and figural analysis. These analyses were conducted using software Atlas.ti.9. All tasks, including the search strategy, data extraction, and analysis, were performed by the first and second authors.

### 3.1. Search Strategy

The first online search was conducted in November 2023, covering studies from the past decade. To ensure the inclusion of the most recent studies, the search was updated in January and May 2024, focusing on publications between January 2014 and April 2024. The reviewer independently searched three electronic databases: Web of Science, Scopus, and ScienceDirect. Additionally, a manual search was performed using Google Scholar to review the references of the complete full-text content. Three key variables were used to generate the keywords from the databases, including the exposure (digital wearable products [keywords] and their technical functions [keywords], outcome (consumer acceptance [keywords], and control or moderator (consumer behavior [keywords]). Table 1 shows the details of the search string and keywords used in the current systematic review. Truncation, Boolean operators, parentheses, wildcards, and quotation marks were applied to refine the search results. The search string was utilized for all fields. Figure 2 illustrates the PRISMA flow diagram depicting the steps of the systematic literature search.

### 3.2. Inclusion and Exclusion Criteria

The study’s inclusion and exclusion criteria were as follows: (a) Publication year and language, including only studies published between 2014 and 2024 in the English language. Studies published before 2014 or in a language other than English were excluded. Based on the existing literature, the field of smart and digital wearable technology (such as smartwatches and fitness trackers) experienced significant growth and innovation in the mid-2010s (around 2014) [25,50]. (b) Study type: including only empirical research articles (quantitative, qualitative, or mixed methods) published in peer-reviewed H-index journals. Review articles, conference papers and proceedings, books, reports, or other text materials not published in peer-reviewed, indexed journals were excluded to ensure the quality and reliability of the selected studies [40,41]. (c) Study scope: Selected studies must include results on the technical function (factors) of DWPs and the context of consumer acceptance and/or human perceptions and outcomes. Studies that did not address at least one result on the technical functions of DWPs were excluded to ensure relevance to the research questions. (d) Target group: The sample of the studies must include adult individuals aged 18 years and over. This age range is targeted due to their higher propensity to adopt new technologies and their significant representation in the consumer market for DWPs [25,50]. The inclusion and exclusion criteria were applied in different screening and identification phases of this systematic review using the Mendeley Desktop, version 1.19.8.

### 3.3. Study Selection

In the first stage of PRISMA (identification), two reviewers (Author 1 and Author 2) independently searched the selected databases using the Electronic Management Research Library Database of the two reviewers’ affiliated universities. They imported the retrieved records from all fields into Mendeley Desktop. The search was limited to English-language articles published between 2014 and 2024 that focused on the technical functions of DWPs. Duplicate records were identified and removed at this stage. In the second stage (screening), the two reviewers screened the imported records based on the type, year of publication, and language. Subsequently, the titles and abstracts were further screened based on the study scope criteria. Studies were included if they addressed the technical functions of DWPs in the context of consumer acceptance and/or human perception and outcomes. Articles selected by at least one reviewer were retained for further assessment. In the third stage (eligibility), the selected articles were retrieved in full text and evaluated based on their relevance to the scope, type of participants, methodology, and quality appraisal of the study. A manual reference search was also conducted using Google Scholar to identify any additional relevant studies. At the last step, the two independent reviewers thoroughly debated the selected articles and agreed on their inclusion; any disagreements regarding study inclusion were resolved through discussion with the third reviewer (Author 3). A summary table was created for the selected full-text articles to present the data from each study (see Appendix A and Supplementary Information, Table S1). The data analysis and synthesis were conducted by the first and second reviewers (Author 1 and Author 2) using Atlas.ti.9 and VOSviewer 1.6.20 software. All authors approved the procedures followed in the search protocol.

### 3.4. Data Extraction, Analysis, and Synthesis

The reviewer (Author 1) extracted a summary of the main information, and all relevant data from the selected articles that could be related to the types of DWPs, technical functions of DWPs, their possible influence on consumer acceptance, and the other related factors (consumer behavior and DWPs related outcomes) and data extraction process were discussed with Authors 2 and 3. The data extraction recheck was performed by Author 2. The data extraction was performed on the final selected papers using a specific extraction framework (shown in the Supplementary Information, Tables S1 and S2) developed by the first and second reviewers (Author 1 and Author 2). The data extraction was performed using Mendeley Desktop, version 1.19.8, and Microsoft Excel 365 [41]. For the primary data and to ensure that all of the themes, categories, and factors (codes) analyzed were significantly related to the systematic review topic, the data extraction was developed in line with the definitions of the conceptual frameworks pertinent to the DWPs consumer acceptance model, as shown in Figure 1.

The first step of the data extraction and analysis was conducting a descriptive bibliometric analysis on the key information of the selected studies (such as year, source, methodology, and country of the selected studies). Besides, a summary table was generated to show the main information of the selected studies (such as title and objective of the study, country and source of the study, wearable type, methodology, and results, see Appendix A, Table A1). In the second step of the analysis, a bibliometric analysis was also conducted on the full-text selected papers to identify the key knowledge that helped to identify the specific themes, categories, and codes related to the review topic, in turn helping in conducting valid thematic and descriptive analysis and synthesis for the systematic review.

The bibliometric analysis was conducted on 38 full-text selected papers using co-authorship and keyword co-occurrence networks for the author and keyword links analysis (using total strength links, occurrences, and frequency). This analytical approach is valuable as it identifies common viewpoints among the publications and authors, therefore guiding in determining the topic themes, related terms, and knowledge structures. For the graphical mapping of keyword co-occurrence networks, network theory was employed, with clustering methods determined by Waltman et al. [42]. All calculations were performed using Microsoft Excel and VOSviewer version 1.6.20 [43].

The last step of the analysis was conducting a descriptive thematic analysis (extracting and synthesizing the data based on the theme, category, and codes and describing the extracted data using frequency, tabular, and figurative analysis) on the key themes, categories, and codes identified from the previous steps [41].

Therefore, based on the theoretical model of the review and the bibliometric analysis results and in line with the research questions, five main themes were identified for data extraction and data analysis: (a) DWPs, (b) technical functions of DWPs, (c) consumer acceptance, (d) user behavior in the consumer acceptance model, and (d) perceived outcomes of the consumer acceptance model. The descriptive analysis of the first theme helped to answer the first question of this systematic review; the second, third, and fourth themes aided in answering the second question. Meanwhile, the last theme guided in answering the third research question.

The first theme (DWPs) was described in eight codes related to the types of DWPs and their connections to the categories and codes of technical functions (second theme), consumer acceptance (third theme), and user behavior (fourth theme). The second theme (technical functions of DWPs) was described in four key categories and 20 codes. The four categories were wearable technology (including PU and PEOU), appearance and design, biomimetic innovation, and security and privacy. Biomimetic innovation is identified in the reviewed studies based on explicit descriptions of nature-inspired principles, structures, or functions, such as biomimetic designs, self-healing materials, skin-mimicking structures or surfaces, thermal regulation, or bio-inspired adaptive structures or systems. The data extracted from the selected studies are based on explicit mention of these terms related to bio-inspired terminology and functional or other design features that replicate biological properties. The third theme (consumer acceptance) was used to describe the concept of consumer acceptance in the DWPs context and their related factors in line with the second and fourth themes. The fourth theme (user behavior in the consumer acceptance model) was discussed in four key codes, including personal factor, social factor, attitude of use, and behavioral intention. It also addressed the relationship between the user behavior codes and the technical functions of DWPs in the consumer acceptance mode. The fifth theme (perceived outcomes of the consumer acceptance model) was described in five key categories and eight codes; the five categories included health and fitness, enjoyment, social value, biomimicry application, and market growth.

Again, the key themes and categories were identified based on the initial bibliometric analysis conducted before the primary thematic analysis in this systematic review; for details, check Section 4.2. (the co-authorship and keyword co-occurrence network).

### 3.5. Study Quality Appraisal

The quality appraisal or quality assessment of the included studies was independently conducted by the first and second reviewers (Author 1 and Author 1) using a standardized evaluation framework, the Newcastle–Ottawa Scale (NOS). The impartial reviewer (Author 3) contributed to resolving any disagreements and made the final decision on the included articles through a verification check using NOS. The Newcastle–Ottawa Scale (NOS) is an eight-item quality assessment checklist used to assess the quality of non-randomized studies in systematic reviews and meta-analyses [41,44]. The Newcastle–Ottawa Scale (NOS) was used to evaluate the risk of bias in selected studies, focusing on assessing three themes: (a) study selection: four factors, (b) study comparability: one factor, and (c) study outcomes: two factors (Supplementary Information 4, Table S4). Studies with a low risk of bias (NOS score from 6 to 7 points) were included in the review. Studies with a high risk of bias (NOS score from 4 to 5 points) and a very high risk of bias (NOS score from 0 to 3 points) were excluded [44]. Detailed quality assessment criteria are outlined in Supplementary Information, Table S4.

## 4. Results

A total of 1209 records were identified through the search utilizing the chosen databases. A manual search of the reference lists of the selected full-text papers and Google Scholar added seven (*n* = 7) additional articles. After removing duplicate studies and including only peer-reviewed journal articles published in the English language from 2014 to 2024, a total of (*n* = 738) papers were retained. However, (*n* = 348) publications were rejected after title screening, and (*n* = 189) were declined following abstract screening, as they were considered ineligible due to the unsuitable scope of the studies (*n* = 422) or the unsuitable sample of the study (*n* = 115). In the eligibility phase, the two reviewers meticulously examined a total of (*n* = 201) full-text papers. Of them, (*n* = 38) full-text articles were included in the thematic and descriptive analysis, while (*n* = 163) articles were excluded. The grounds for exclusion were as follows: (*n* = 56) articles were review studies, (*n* = 18) articles involved samples that were not aged 18 years and above, (*n* = 84) articles did not include any results on DWPs and consumer acceptance or human interaction, and (*n* = 5) studies did not meet the required quality standards (NOS total quality score less than 6). The rejected articles were deficient in outcome data, precise exposure identification, appropriate control of confounding variables, or sufficient sample size. Figure 2 presents a PRISMA flowchart that delineates the selection procedure. The outcomes were synthesized and analyzed following the five identified themes.

### 4.1. Characteristics of the Selected Study

**Figure 3:**
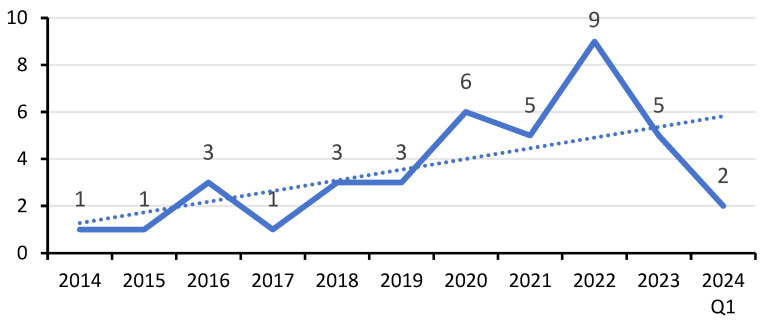
Number of articles by year of publication.

Figure 3 illustrates the annual evolution of published studies within the period under investigation. The mean year of publication is (2020.3 ± 2.6), indicating that this is an emerging field of research. The frequency of the publication year showed that most of the selected papers were published in 2023 (*n* = 9, 23.68%) studies, followed by 2020 (*n* = 6, 15.78%) studies, and 2021 and 2023 (*n* = 5, 13.15%) studies each. The data reveal a gradual increase in studies on this topic beginning around 2017.

Regarding the studies’ sources, the 38 publications included in the review were published in (*n* = 31) sources. The top five sources with the highest number of publications were: Technology in Society (*n* = 3 references), Computers in Human Behavior (*n* = 3 references), Digital Health (*n* = 2 references), International Journal of Human–Computer Interaction (*n* = 2 references), and npj Digital Medicine (*n* = 2 references), see Table 2. Table 2 also shows that the pioneer publisher in the field was Sensors from MDPI, with a publication in early 2014; meanwhile, the most recent publication was published in Frontiers in Public Health.

**Figure 4:**
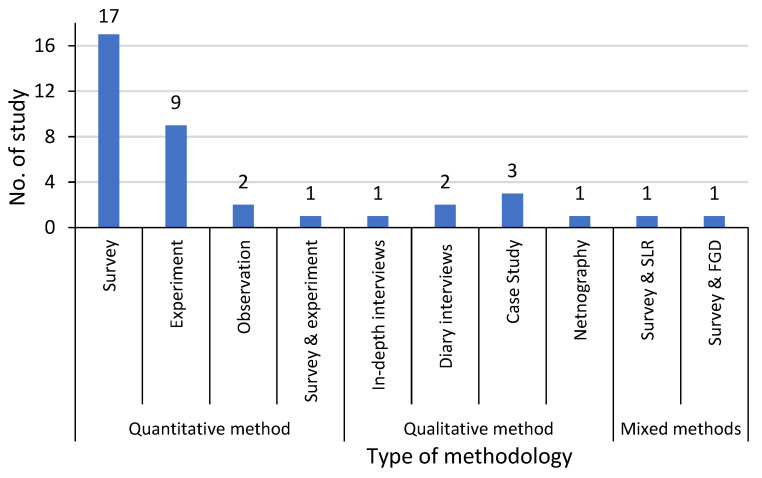
Distribution of research strategies.

Figure 4 illustrates the distribution of methodologies utilized in the 38 selected papers. Most of the selected studies (*n* = 29, 76.31%) had adopted quantitative methods, applying a variety of data collection methods, including questionnaire survey (*n* = 17, 44.73%), experimental approaches (*n* = 9, 23.68%), observation study (*n* = 2, 5.26%), and mixed quantitative survey and experimental approaches (*n* = 1, 2.63%) study, see Figure 3 and Appendix A, Table A1. These were followed by seven qualitative studies (*n* = 7; 18.42%) that applied four main qualitative methods, including case study and interviews (*n* = 3, 7.89%), diary interviews (*n* = 2, 5.26%), netnography (*n* = 1, 2.63%), and in-depth expert interviews (*n* = 1, 2.63%) study. Two studies were mixed methods (*n* = 2; 5.26%) using a systematic review and questionnaire survey (*n* = 1, 2.63%) and online survey and focus group discussions (*n* = 1, 2.63%) study. Research strategy is critical in outlining the general approach researchers take to address their research questions.

**Figure 5:**
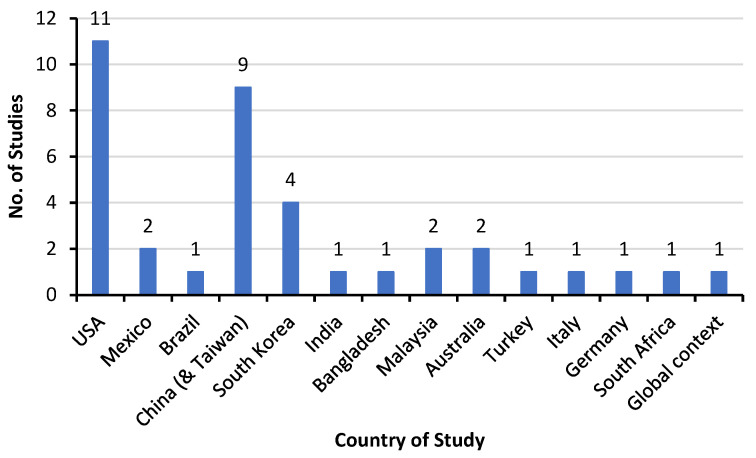
Countries with the most publications.

Figure 5 shows the distribution of the countries where the selected studies were conducted. Overall, the selected studies were conducted in (*n* = 13) countries around the world. Most of the selected studies have been done in the USA (*n* = 11, 28.94%) studies, followed by China (*n* = 9, 23.68%) studies, South Korea (*n* = 4, 10.52%) studies, Australia and Malaysia (*n* = 2, 5.26%) studies each, Mexico, Brazil, India, Bangladesh, Turkey, Italy, Germany, and South Africa (*n* = 1, 2.63%) study each. Besides, one of these studies was conducted in a global context, including Europe, America, Asia, and Australia (*n* = 1, 2.63%) study, see Figure 5.

### 4.2. The Co-Authorship and Keyword Co-Occurrence Network

Scientific mapping with VOSviewer software, version 1.6.20, was used to analyze the keyword co-occurrence network in the selected papers, revealing core knowledge and intellectual structures in the field. This approach is crucial for identifying the more specific themes and topics covered in the included studies [8]. Using VOSviewer, a group visualization of the keywords with the highest occurrence was generated based on the included full-text studies. The results show that in the 38 studies analyzed, there were a total of 160 keywords, of which 10 domain clusters were identified, connected by 462 links, with a total link strength (TLS) of 486. Each cluster was differentiated by color and node size, indicating keyword occurrences, representing the key topics (themes) in the field.

**Figure 6:**
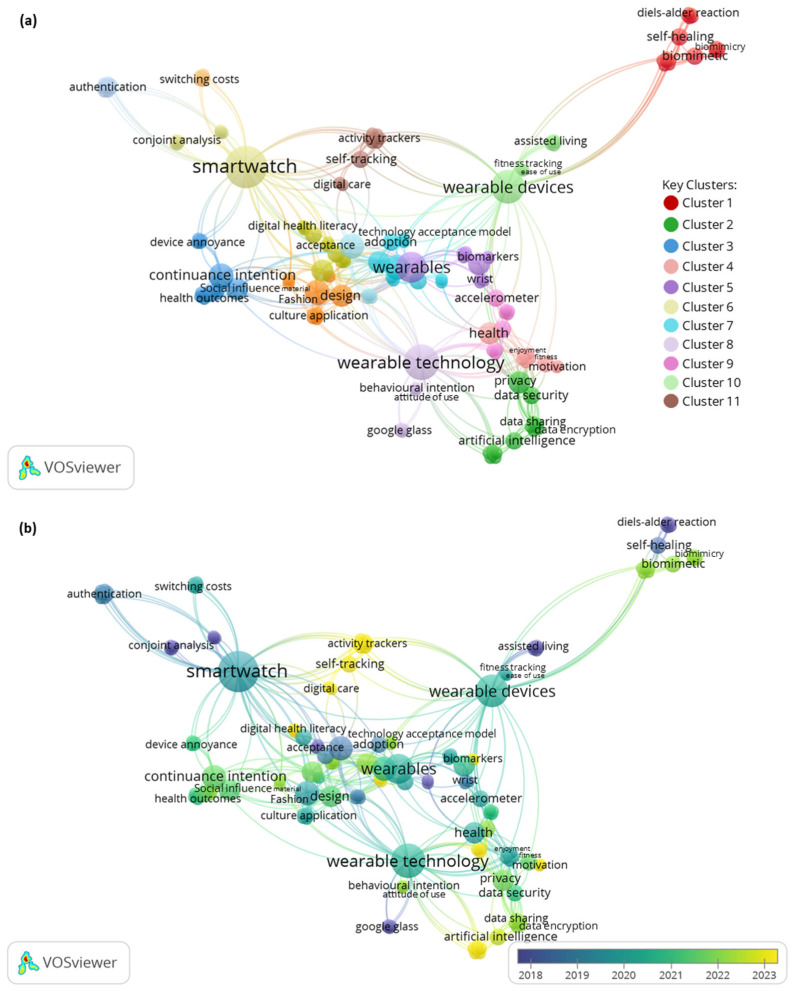
Keyword co-occurrence network. (**a**) Network visualization, (**b**) overlay visualization. (Source: Author records using VOSviewer, version 1.6.20).

The keyword co-occurrence network map showed a visualization of the terms (keywords) that appear most frequently in each cluster. The keywords highlighted in more than (*n* = 10) links are listed in Table 3 and included four key themes: (a) keywords related to DWPs and their functions; (b) keywords on consumer acceptance; (c) keywords related to users’ behavior; and (e) keywords on perceived outcomes, among other related terms (see Figure 6). This approach is guided to better understand the categories and codes that will be targeted in the data extraction and synthesis of the thematic analysis for the following sections.

More specifically, Cluster 1 (red) showed the new trends in biomimetic innovation technology represented in the wearable sensors and motion sensors and their impact on biomimicry trends and human self-healing and health [31,77,89]. The distribution of these keywords based on the year of publication showed that the self-healing wearable sensors were a frequent topic in 2018 [77], paving the way for studies that focused primarily on biomimetic innovation and their relations to consumer intention between 2021 and 2024 [31,89]. Cluster 2 (green) showed the key insights into the technical function (privacy, security, data encryption, and data protection) of the DWPs; it also highlighted the words that artificial intelligence linked to the consumer intentions [35,48,50,58]. The overlay visualization for the keyword publication time showed that the most recent research is emphasizing healthcare biomarkers and trackers [35]. It also shows another emerging topic, artificial intelligence (AI), connected to the wearable technology model. Cluster 3 (blue) highlighted the role of social influence and social capital in the acceptance model (mentioned as continuance intention) of the digital devices; this topic was discussed between 2020 and 2022. This cluster was significantly connected with Cluster 6 (beige) and Cluster 8 (bright purple). Cluster 4 (nude) addressed the acceptance of technology and its connection to perceived health [73]. It also highlighted the privacy and security factors.

Cluster 5 (purple), Cluster 6 (beige), Cluster 9 (pink), and Cluster 10 (bright green) highlighted the types (smartwatch, activity tracker, activity monitor, accelerometer, sensors, and so on) and functions (health monitoring (PU), fitness tracking (PU), ease of use (PEOU), and other technology factors) of DWPs and their relationship with the individuals’ acceptance, affected by their behaviors and social influence [58,64]. Cluster 7 (Ferozi) and Cluster 8 (bright purple) showed keywords related to consumer acceptance (acceptance, TAM, adaptation, and so on) and consumer behavior (behavior intention and attitudes). Cluster 8 (bright purple) also highlighted the keywords related to the types of DWPs, such as Google Glass.

Therefore, from the analysis of the keyword co-occurrence network map (Figure 6), the key themes of this review included the following: (a) DWPs based on the keywords from Clusters 5, 6, and 10. (b) technical functions of DWPs as discussed in Clusters 1, 2, 7, and 10. (c) Consumer acceptance, as highlighted clearly in Clusters 3 and 7. (d) User behavior in the consumer acceptance model based on the keywords in Clusters 3 and 8. (e) Perceived outcomes of the consumer acceptance model in line with the keywords in Clusters 1 and 4. The keywords’ publication time in the overlay visualization showed that the trend research in the field focuses on biomimetic innovation of the digital wearables and bio wearables (sensors) and their health outcomes [28], see Figure 6b.

VOSviewer software was also used to analyze the author and co-authorship network in the selected papers, in order to highlight the most impactful authors in the field of the DWPs acceptance model. The analysis revealed that 166 authors participated in the publication of the 38 full-text papers, with an average of 4.36 authors per paper. The analysis of the co-authorship was based on the number of documents for each author in the 38 selected papers and the co-authorship links with other authors in the papers. The greater TLS represents strong co-authorship links with other authors in the paper(s). The minimum number of documents per author was set to one in the chosen threshold in the VOSviewer author analysis. The co-authorship analysis showed that there were only two authors who contributed to more than one paper: Seongcheol, K. (two documents, two TLS) [51,61], who focused on consumer behavior and attitude towards wearable products (smartwatch) contributed to the topics of Cluster 7 in the keyword co-occurrence network, and Lee, E.J. (two documents, no TLS) [70,73], who studied the technical functions (such as design and visual aesthetics) of digital wearables and their perceived enjoyment outcome in line with Cluster 7 above. Meanwhile, in terms of co-authorship links, the authors of the largest-scale studies were Beck, L., with a total of 15 links (15 TLS) [49], with a research focus on wrist-worn digital devices for sleep tracking; Colbert, E. (13 TLS) [30], with a study focusing on intention to wearable biosensors and health outcomes as shown in line with Cluster 8 topics; and Berry, J. (10 TLS) who studied the medical wearable device for physical activity measures [58] as highlighted in Cluster 5 above.

### 4.3. DWPs and the Acceptance Model

This section discusses the first theme of the “DWPs” and was discussed in all of the selected full-text papers (*n* = 38, 100%). These studies primarily investigate the functionality, technological challenges, usability, and consumer intention related to the DWPs. The analysis of the 38 studies identified eight (*n* = 8) types of DWPs, taking into account that some of the selected studies included more than one type of DWPs. The identified DWPs included smartwatch in (*n* = 20, 52.26%) studies [46,47,48,51,52,54,55,59,60,61,63,64,65,66,67,68,69,70,71,74], smart medical devices and robotics in (*n* = 8, 21.05%) studies, [28,30,31,49,58,76,77,78], wearable fitness devices in (*n* = 7, 18.42%) studies [48,66,67,68,73,74,76], wearable fashions [30,31,58,78] and general wearable technology [53,56,72,75] in (*n* = 4, 10.52%) studies each, smart glass in (*n* = 2, 5.26%) studies [62,67], and wristwatch accelerometer [50] and sports wearables [1] in (*n* = 1, 2.63%) study each. Smart medical devices and robotics include various types of products, such as wearable sensors and climbing robots [28], Scanadu Scout, iHealth-finger [30], wearable electrodes [31], wrist-worn devices (e.g., GeneActiv) [49], ActiGraph Insight Watch, ankle-worn activity monitor, Modus StepWatch [58], WIoMT, biosensors [76], motion sensors [77], strain sensors [78]. The mentioned examples of wearable fitness devices were Fitbit, Apple smartwatch [48], and fitness trackers [74]. Both smartwatches and wearable fitness devices were related to four categories of DWPs technical functions, including PU of wearable technology, PEOU of wearable technology, appearance and design, and security and privacy. Smartwatch was also highly connected with the acceptance model by (*n* = 12) codes and user behavior by (*n* = 13) codes (see Table 4). Wearable fitness devices were associated with the acceptance model and user behavior by (*n* = 6) codes each.

Smart medical devices and robotics were connected to four categories of DWPs technical functions, including PU of wearable technology, PEOU of wearable technology, security and privacy, and biomimetic innovation, and correlated to the acceptance model by (*n* = 10) codes and user behavior by (*n* = 6) codes. Wearable fashion products, such as clothing and jewelry, were also related to four categories of DWPs technical functions, including PU of wearable technology, PEOU of wearable technology, appearance and design, and biomimetic innovation. They connected with the acceptance model by (*n* = 4) codes and user behavior by (*n* = 5) codes.

**Figure 7:**
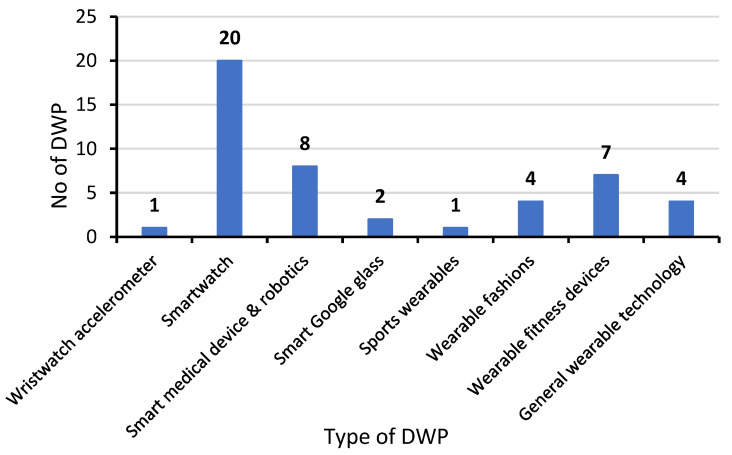
Types and frequency of DWPs based on the selected studies.

General wearable technology was related to three categories of DWPs technical functions, including PU of wearable technology, appearance and design, and security and privacy. They directly affected the acceptance model and user behavior by (*n* = 2) codes each. Other wearable technologies (such as smart Google glass, wristwatch accelerometer, and sports wearables) were also linked to various categories of DWP technical functions and related to the acceptance model and user behavior (see Table 4). Therefore, the technical function of these different types of DWPs significantly shaped the model of user acceptance and behavioral engagement (see Figure 7).

### 4.4. Technical Functions of DWPs

This section discusses the second theme, “technical functions of DWPs,” and was discussed in all of the selected full-text papers (*n* = 38). The analysis of the 38 full-text papers revealed that DWPs offer a range of technical functions beyond just displaying time, including (1) wearable technology factors mentioned in (*n* = 27, 71.05%) studies as (a) PU, in (*n* = 19, 48.71%) studies [1,30,46,47,48,49,50,53,54,56,57,58,61,63,64,65,66,68] and (b) PEOU, in (*n* = 10, 26.31%) studies [1,31,52,59,60,62,67,68,69,77]. (2) Appearance and design mentioned in (*n* = 8, 21.05%) selected studies [51,56,70,71,72,73,89]. (3) Biomimetic innovation factor mentioned in (*n* = 6, 15.78%) studies [28,31,57,77,78,89]. (4) Security and privacy factors mentioned in (*n* = 3, 7.89%) studies [74,75,76], see Table 5.

PU in DWPs denotes the extent to which a user believes the digital product will assist in achieving their job performance or daily activities and improving their overall quality of life. It is a critical factor in promoting users’ data on health, fitness, data management, and lifestyle [30,46,47,48,49,53,54,61,64,66,68]. PU is represented in six (6) codes: “health monitoring” in (*n* = 7, 18.42%) studies [30,46,49,50,53,58,65], “fitness tracking” in (*n* = 6, 15.78%) studies [1,48,54,64,65,68], “lifestyle monitoring” in (*n* = 5, 13.15%) studies [47,55,56,63,66], “data feedback” in (*n* = 4, 10.52%) studies [30,46,48,64], “AI” in (*n* = 2, 5.26%) studies [49,57], and “productivity” in (*n* = 1, 2.63%) study [61].

Meanwhile, PEOU denotes the extent to which an individual perceives that employing a specific wearable technology necessitates minimal effort. PEOU specifically includes users’ perceptions regarding the simplicity and intuitiveness of a wearable device’s interface, the clarity of its capabilities, and the overall ease of its usage [60,67,68]. PEOU is represented in two (2) codes in the selected studies: “ease of use” in (*n* = 8, 21.05%) studies [1,52,59,60,62,67,68,69] and “wearing comfort” in (*n* = 2, 5.26%) studies [31,77].

Appearance and design refer to the DWPs’ appearance, user interface, and user interface, which directly affect the comfort, attractiveness, and continued use of a wearable device, which in turn affects users’ adherence and engagement [70,89]. It is represented in three (3) codes: “visual appeal” in (*n* = 6, 15.78%) studies [51,55,56,70,72,73], “fashion fusion” in (*n* = 3, 7.89%) studies [55,70,89], and “UI aesthetics” in (*n* = 1, 2.63%) study [71]. The user interface significantly influences users’ emotional and practical responses to the DWPs. Consequently, appearance and design are not solely aesthetic characteristics but essential technical functions that connect the technological capabilities of DWPs with user-centered design concepts, hence fostering sustained user engagement [51,56,70].

Biomimetic innovation (or biomimicry), the technique of learning from and replicating nature’s designs, plays a key part in the technological functionality of DWPs. It refers to the application of biological principles and structures to the design and development of wearable technologies [12,23]. These products might utilize various biomimicry principles represented in four (4) codes, such as “skin-mimicking structure” in (*n* = 3, 7.89%) studies [28,31,89], “self-healing structure” and “biomimetic structure” in (*n* = 2, 5.26%) studies each [28,57,77], and “thermal regulation structure” in (*n* = 1, 2.63%) study [78].

Although DWPs deliver convenience and health monitoring, they present security and privacy concerns due to its inherent vulnerabilities and the sensitive data it collects. Therefore, security and privacy are key technical functions in DWPs that include four (4) codes mentioned in the selected studies: “data encryption,” “anonymity protection,” “privacy policy,” mentioned in (*n* = 2, 5.26%) studies each [74,75,76], and “identity authentication” in (*n* = 1, 2.63%) study [76].

Therefore, the technical functions in DWPs can be defined as a set of operational and design-related aspects that directly influence the product’s performance, usability, security, and biomimetic capabilities. More specifically, the technical functions in DWPs can be described in four key categories, including wearable technology factors (PU and PEOU), appearance and design aesthetics, security and privacy, and biomimetic innovation. These functionalities are vital for boosting user interaction, acceptability, and long-term engagement with the device.

### 4.5. Consumer Acceptance and Related Factors

This section discusses the third theme, “consumer acceptance,” which was discussed directly in (*n* = 22, 57.90%) of the selected full-text papers. Other (*n* = 16) studies have examined the DWPs in the context of human perceptions and benefits. However, this section will focus on the results related to consumer acceptance only in line with the context of this study. Consumer acceptance in the context of DWPs refers to the degree to which individuals intend to adopt, use, or purchase wearable technology based on their attitudes, intentions, and perceptions of the product’s technical functionality. The analysis of the full-text papers showed that the consumer acceptance was analyzed in the majority of the selected studies and covers three core dimensions (codes): “consumer attitude” [51], “intention to use/purchase” [1,30,52,53,54,55,56,60,61,62,63,64,65,68,70,71,76,77,89], and “technology acceptance” [59,69].

The analysis in Table 5 shows that all of the technical functionality categories are predicting the consumer acceptance model through various codes. Many of the selected studies showed that PU and PEOU of wearable technology were strong predictors of consumer acceptance through all of their codes: PU in (*n* = 11, 28.94%) studies [1,30,49,53,54,56,61,63,64,65,68] and PEOU in (*n* = 8, 21.05%) studies [1,52,59,60,62,68,69,77]. Four (*n* = 4, 10.52%) of the analyzed studies showed that all codes on appearance and design predicted consumer acceptance [51,55,56,70]. Two (*n* = 2, 5.26%) of the studies showed that biomimetic innovation predicted the consumer acceptance through three codes (*n* = 3) (self-healing structure, skin-mimicking structure, and thermal regulation structure) [77,89]. Security and privacy were also predictors of consumer acceptance through their four codes (*n* = 4) as shown in Thapa et al. [76] study, see Table 5. Furthermore, sixteen (*n* = 16) of the selected studies highlighted that user behavior significantly correlated (predictor or moderator) to the consumer acceptance model through four codes: “personal factor,” “social factor,” “attitude of use,” and “behavioral intention” [30,51,53,54,55,56,60,61,62,64,65,69,70,71,76,89]. Therefore, a thorough understanding of consumer acceptance must encompass both the technological functions of DWPs and the broader context of user experiences and anticipations.

### 4.6. User Behavior in the Consumer Acceptance Model

The fourth theme of user behavior in the consumer acceptance model constitutes a crucial area of investigation in digital wearable technology research, concentrating on individuals’ intention to utilize or persist in using these technologies. User behavior is another key dimension in the DWPs consumer acceptance model. The SLR and descriptive analysis showed that user behavior was highlighted in (*n* = 19, 50%) studies that include four aspects (codes): “personal factor,” in (*n* = 11, 28.94%) studies [30,46,51,53,54,60,61,69,70,71,76], “social factor” in (*n* = 5, 13.15%) studies [55,62,64,65,70], “attitude of use” in (*n* = 5, 13.15%) studies [53,56,57,61,64], and “behavioral intention” in (*n* = 5, 13.15%) studies [48,53,61,62,89].

These various aspects (codes) of user behavior usually play a critical moderator role in the relationship between factors (categories and codes) of technical functions and the consumer acceptance variable in the DWPs acceptance model. Table 5 shows the multidimensional interactions between the technical functional and user behaviors of DWPs, highlighting how the technical functional aspects, such as PU, PEOU, appearance and design, biomimetic innovation, and security and privacy, influence consumers’ acceptance through their behavior. It shows that all of the technical functionality categories were affected by the four aspects of user behavior. The descriptive analysis showed that user behavior was a moderator in the consumer acceptance model and the PU of wearable technology in (*n* = 9, 23.68%) studies [30,53,54,55,56,57,61,64,65], PEOU of wearable technology in (*n* = 3, 7.89%) studies [60,62,69], appearance and design in (*n* = 6, 15.78%) studies [51,55,56,70,71,89], biomimetic innovation in (*n* = 1, 2.63%) studies [89], and security and privacy in (*n* = 1, 2.63%) study [76]. User behavior was also a direct factor affecting intention to PU of wearable technology in (*n* = 2, 5.26%) studies [46,48] and biomimetic innovation in (*n* = 1, 2.63%) studies [57].

Furthermore, as mentioned above, various codes of user behavior were key moderators in the DWPs consumer acceptance model: “personal factor” highlighted in (*n* = 9, 23.68%) studies as a potential moderator in the consumer acceptance model [30,51,53,54,60,61,69,71,76]. “Social factor” in (*n* = 5, 13.15%) studies [55,62,64,65], “behavioral intention” in (*n* = 4, 10.52%) studies [53,61,62,89], and “attitude of use” in (*n* = 4, 10.52%) studies [53,56,64] were potential moderators in the relationship between technical function and consumer acceptance in the DWPs acceptance model. Therefore, the various aspects of technical functions directly affect users’ behaviors and intentions, in turn affecting consumer acceptance. At the same time, the user behavior factors moderate the relationship between technical functions and consumer acceptance.

### 4.7. Perceived Outcomes of the Consumer Acceptance Model

This section discusses the fifth theme, “perceived outcomes of the consumer acceptance model,” and was discussed in (*n* = 31, 81.57%) of the selected full-text papers, see Table 6. The term “perceived outcomes” is used to describe the key benefits and contributions of the DWPs in the context of the consumer acceptance model. Table 6 shows the perceived outcomes (contributions) of the DWPs, including (1) health and fitness mentioned in (*n* = 23, 60.52%) studies as “health motivation” and “fitness performance” [1,28,30,31,46,47,48,49,52,53,59,60,61,65,66,67,68,69,70,71,74,76,89]; (2) enjoyment outcome represented in (*n* = 8, 21.05%) studies in two codes: “hedonic pleasure” and “perceived enjoyment” [54,56,61,64,70,71,78,89]. (3) Social value highlighted in (*n* = 5, 13.15%) studies as “social recognition” and “socialization.” [30,64,65,70,71]. (4) Biomimicry application was also highlighted in (*n* = 5; 13.15%) studies as “bio-inspired technology” and “self-healing” [28,31,77,78,89]. (5) Market growth mentioned in (*n* = 4, 10.52%) studies as a key outcome (result) in the DWPs model [62,72,75,89].

The health and fitness represented the most highlighted outcome related to the technical functions of DWPs, related to PU of wearable technology (in *n* = 11 studies, 28.94%) [1,30,46,47,48,49,53,61,65,66,68], PEOU of wearable technology (in *n* = 7 studies, 18.42%) [31,52,59,60,67,68,69], appearance and design (*n* = 3 studies, 7.89%) [70,71,89], security and privacy (*n* = 2 studies, 5.26%) [74,76], and biomimetic innovation (*n* = 1 study, 2.63%) [28]. The analysis also shows that health and fitness are the most outcome-related to the consumer acceptance highlighted in (*n* = 11, 28.94%) studies [1,30,52,53,59,60,61,65,68,69,70,71,76,89], see Table 6.

The enjoyment outcome was significantly related to three technical functions, including PU of wearable technology in (*n* = 4, 10.52%) studies [54,56,61,64], appearance and design (*n* = 4, 10.52%) studies [56,70,71,89], and biomimetic innovation (*n* = 1, 2.63%) study [78]. The emotional outcome was found to be related to consumer acceptance in (*n* = 6, 15.78%) studies [54,56,61,64,70,71,78,89]. Social value was correlated to two technical functions, including PU of wearable technology (*n* = 3, 7.89%) studies [30,64,65] and appearance and design (*n* = 2, 5.26%) studies [70,71]. Besides, social value was correlated to consumer acceptance in (*n* = 5, 13.15%) studies [30,64,65,70,71]. Meanwhile, biomimicry was significantly correlated to three technical functions, including PU of wearable technology (*n* = 3, 7.89%) studies [28,77,78], PEOU of wearable technology (*n* = 2,5.26%) studies [31,77], and biomimetic innovation (*n* = 5, 13.15%) studies [28,31,77,78,89]. Biomimicry was a related outcome to consumer acceptance in (*n* = 2, 5.26%) studies [77,89]. Market growth was connected to four technical functions: PEOU of wearable technology (*n* = 1, 2.63%) study [62], appearance and design (*n* = 2, 5.26%) studies [72,89], biomimetic innovation (*n* = 1, 2.63%) study [89], security and privacy (*n* = 1, 2.63%) study [75], and consumer acceptance (*n* = 1, 2.63%) study [62]. Overall, different categories of DWPs’ technical functions were related to varying codes of perceived outcomes, reflecting the significant role of technical functions in the DWPs acceptance model.

## 5. Discussion

**Figure 8:**
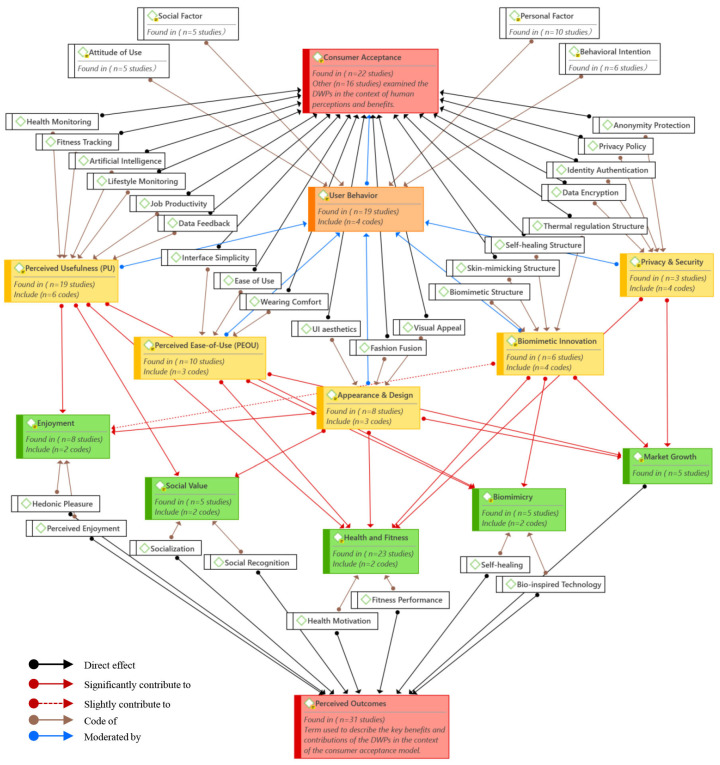
DWPs in consumer acceptance model using Atlas.ti.9.

In the last ten years, the domain of DWPs and robotics has undergone swift growth, propelled by advancements in sensor technologies, bionic and biomimetic innovations, wireless communication, and artificial intelligence [5,8]. This increase has converted wearable devices from specialized fitness gadgets into ubiquitous instruments incorporated into healthcare, sports, fashion, and everyday life [13,16,90,91]. The expanded interest in research and development and production by technology firms has profoundly impacted the progression of consumer acceptance models worldwide [5,6,92]. Therefore, due to this increase in research and industry production in the field of digital wearable technologies, it was considered necessary to conduct a systematic review that would provide a comprehensive understanding of the role of the technical function of DWPs in the consumer acceptance model, see Figure 8.

In line with the research questions, the objective of this systematic review was to identify the different types of DWPs and their related emergent technical functions in the consumer acceptance model; to understand the role of emergent technical functions in influencing consumers’ acceptance and behavior of DWPs; and to determine the related outcomes of DWPs and their emergent technical functions in the consumer acceptance model. To answer the research questions, a comprehensive systematic literature review with a bibliometric, descriptive, and thematic analysis was conducted on the full-text of 38 indexed peer-reviewed journal articles published between 2014 and 2024. The bibliometric analysis was conducted first to provide a general understanding of the topic of WDPs in consumer acceptance. It helped to identify the specific themes and topics covered in the included studies. Therefore, the bibliometric analysis led to identifying five themes that will be studied in the thematic analysis, including (a) DWPs, (b) technical functions of DWPs as discussed, (c) consumer acceptance, (d) user behavior, and (e) perceived outcomes of the consumer acceptance model. However, the bibliometric visualization analysis only focused on keywords and co-authorship analysis, which could not give the whole picture of the knowledge on the topic. Therefore, the further thematic analysis of the systematic review aimed to provide a better understanding of the comprehensive multidimensional perspective of the DWPs in the consumer acceptance model. Additionally, the author and co-authorship network analysis using VOSviewer revealed critical structural characteristics of research collaboration in the domain of DWPs acceptance models. The total of 166 unique authors publishing 38 full-text studies, with an average of 4.36 authors per paper, indicates a moderately collaborative research community, consistent with the interdisciplinary nature of wearable technology research, which often integrates engineering, behavioral sciences, and health domains [67,88,93,94]. However, the results indicate limited collaboration among authors, as they show only two authors contributed to more than one paper within the analyzed dataset [51,61]. This result suggests that research on DWPs and consumer acceptance remains somewhat fragmented, highlighting the need for more collaborative studies to build integrated models and shared frameworks [88,94]. In terms of co-authorship links, three authors are central connectors with the highest TLS, indicating their role in fostering cross-study co-authorship networks [30,49,58]. These studies highlighted the focus on emergent topics in the field, such as wearable biosensors for health monitoring and medical wearables for physical activity measurement [49,58].

Regarding research question one (RQ1), it identified the types of DWPs and the related technical function of each type in the context of consumer acceptance. This systematic review revealed eight significant types of DWPs in the digital consumption market, including smartwatch, medical devices and robotics, wearable fitness devices, wearable fashions, smart glass, wristwatch accelerometer, sports wearables, and other general wearable technology. These different types of DWPs played a pivotal role in shaping the model of consumers’ perceptions, acceptance, and behavioral engagement in the digital products market [13,17,95].

Prior research often grouped the smartwatch, wristwatch accelerometer, sports wearables, and wearable fitness devices under “health tracking wearables,” focusing on health tracking and connectivity as an example of wearable technology in healthcare [22,25]. There were a variety of wearable devices and robotics identified in the review “medical devices and robotics,” such as glucose monitors, robotic exoskeletons, wearable electrodes, and climbing robots, ankle-worn activity monitors, and more, due to their implication in the medical field. However, a previous narrative analysis by Rahman et al. [18] and experimental work by Zhong et al. [21] only focused on the clinical adoption barriers but did not always integrate them into broader consumer digital markets. Yet, the smart medical devices and robotics have been recently used more widely by individuals for healthcare at home. Due to the high demand, the industry has begun developing these products for personalized care and improved medication management by implementing the ability to enable remote monitoring and easy-to-use functions [30,78,96]. Along with the health and medical functionality, the review also highlighted the convergence of wearable technology with the fashion and jewelry sector under the “wearable fashions” [55,89,97].

No longer confined to fitness trackers and medical wearables, smart devices have grown into lifestyle items that mix usefulness with fashion and design, appealing to customers’ intentions [12,61,98]. The review showed that all of these DWPs were related to consumer acceptance and consumer behaviors through one or more of the technical functions of these products. For example, “smartwatches” and “wearable fitness devices” had a significant connection with the consumer acceptance and behavior through the PU, PEOU, appearance and design, and security and privacy, meaning that the consumers had a higher demand for the smartwatches and wearable fitness devices that provide multiple functions and usefulness and ease of use, are well-designed, and offer security and privacy [46,52,55,60,66,74,76,99]. Similarly, Liu et al. [22] and Erzetic et al. [27] discovered that the ease of use, aesthetic appeal, and fashion compatibility of digital wearables influence both purchasing intention and sustained usage. Previous research has also shown that devices that offer adequate data security are more likely to be accepted, especially in health-conscious or data-sensitive sectors [13,34].

Consumers also had more intentions to use “smart medical devices and robotics” that promote usefulness and function (such as health monitoring and AI), ease of use, biomimetic properties (self-healing material and thermal regulation), and security and privacy [28,30,49,77,78,100,101]. The dynamic integration of AI was a fundamental function of the PU in the biotic and assistive robotics [49]. These findings contributed to the Lu et al. [99] study, highlighting that consumers are more inclined to use healthcare wearables when they regard them as effective instruments for continuous health monitoring, particularly in controlling disorders such as diabetes or cardiovascular diseases. The ease of use of these devices significantly affects user behavior; intuitive interfaces, seamless integration, and little user effort were essential for widespread acceptance [51,53]. More recently, developments in biomimetic design, including self-healing materials, skin-mimicking structures, and thermal regulation systems, have begun to shift customer expectations and intentions. According to Chen et al. [28], such bio-inspired elements, like biomimicry, not only improve comfort and wearability but also boost trust in device sustainability and the healing aspect.

Furthermore, consumer interest in wearable fashions (smart clothing and jewelry) was also driven by functionality, usability, design, appearance, and biomimetic innovation of these products. As wearable trends advance, customer preferences continue to be molded by the convergence of design sensibility and innovative material science, underscoring the significance of a multidisciplinary approach to product development [57]. A qualitative study from Taiwan conducted by Chang and Lin [89] suggested that such bio-inspired aspects not only boost functionality but also give wearables an e-skin quality that matches the body’s natural tendencies, improving comfort and perceived sophistication, in turn affecting the wearable acceptance in the consumption markets.

Concerning research question two (RQ2), the thematic analysis revealed that the emergent technical functions of the DWPs can be divided into four functions: (1) wearable technology factors (PU and ease of use), (2) appearance and design, (3) biomimetic innovation, and (4) security and privacy concerns, see Figure 8. These findings align with and extend existing technology adoption theories, such as TAM, while highlighting unique considerations for wearable devices [36]. Regarding the first function, wearable technology, it mirrors the core constructs of TAM and includes two sub-functions: (a) PU, which includes health monitoring, fitness tracking, lifestyle monitoring, data feedback, artificial intelligence (AI), and productivity, and (b) ease of use (PEOU), including ease of use and wearing comfort. The review findings demonstrate that both PU and PEOU serve as significant predictors of consumer acceptance for wearable technologies and digital robotics. AI, an emerging discipline, also played an increasingly vital role as a technological function in wearable items and robots, considerably impacting customer acceptance to embrace and continue using these technologies. It expanded the device intelligence by providing context-aware services, real-time analytics, predictive monitoring, and personalized feedback, which increased the PU of these devices [57,75]. These results were in line with Chun et al. [52], who identified key technological aspects for smartwatches, revealing the importance of health and fitness tracking functions, data synchronization, and AI in promoting consumer intentions towards these DWPs. The findings also contribute to the Ma et al. [29] findings, which revealed that comfort and seamless integration with everyday routines were critical for the long-term usage of multifunctional wearable sensors, indicating that ease of use in wearables must be reconceptualized to include ergonomic elements.

The second function, appearance and design, was reflected in three aspects: visual appeal, fashion fusion, and UI aesthetics. All of these design functions were critical in predicting consumer acceptance. Overall, the appearance and design of DWPs encompass both the physical aesthetics (such as color, form, and material) and the user interface (UI), which play a vital role in determining user comfort, attractiveness, and sustained engagement. These characteristics are not only about visual attractiveness but also relate to the physical and ergonomic qualities that determine how functional an item is worn [70,89]. Notably, Wang et al. [71] showed that various interface elements, including symmetry, visual complexity, and the curvature of display edges, might influence users’ perception of both enjoyment and functionality, which in turn impact their intention to continue using wearable devices.

The third function, biomimetic innovation, also significantly predicted the consumer intention towards the DWPs through three functions, including self-healing structure, skin-mimicking structure, and thermal regulation structure. For instance, self-healing materials designed based on flexible self-healing nanocomposites contribute to expanding the lifetime of sensor devices and empower them with economic and safety attributes, which in turn might enhance users’ needs and increase their acceptance [31,77]. The integration of emergent functions, such as skin-mimicking and biomimetic self-adhesive structures, inspired by the adhesion ability of various organisms in nature, into wearable devices can be achieved by grafting biomimetic intelligence [28,32]. This mechanism contributes to the development of biomimetic innovation in wearable technology, enabling optimization of their usage and, in turn, increasing users’ behavioral intentions in the acceptance model, leading to their biomimicry market growth [31,89]. Biomimetic innovations within wearable technology comprise an emerging and significant research theme, attempting better gadget functioning, comfort, and user acceptance by taking inspiration from biological processes [31]. Besides, the findings supported Pan et al. [32], highlighting that biomimetic wearable sensors, by emulating biological sensory systems like human skin or the fish lateral line system, provide more reliable health and environmental data for users, thereby enhancing user intention to utilize them.

The fourth function of security and privacy concerns included four factors: data encryption, anonymity protection, privacy policy, and identity authentication, which also predicted consumer acceptance. Although DWPs provide a variety of functions, they might pose considerable security and privacy concerns [76,102]. Therefore, each of these functions addresses a specific layer of digital risk mitigation. Data encryption guarantees that health or location information remains secure from interception or misuse during transmission, while anonymity protection dissociates user identities from raw data, thereby safeguarding user profiles [74,76]. For instance, Sivakumar et al. [80] advocate for a comprehensive strategy to mitigate privacy issues while optimizing the health advantages of wearable devices; they underscore the necessity for ethical guidelines that take into account human decision-making behaviors, enabling users to make informed choices that correspond with their privacy preferences and health objectives.

At the same time, this review also discussed user behavior as a moderator in the consumer acceptance of DWPs. It defined user behavior in four key factors, including the personal factor (referring to the demographic variables), social factor (such as social influence and social capital), attitude of use, and behavioral intention (individual’s readiness or plan to perform a specific behavior). The findings of the systematic review showed that these behaviors play a critical moderator role in the relationship between the technical functions and the consumer acceptance in the DWPs acceptance model. Similarly, Wu et al. [60] conducted empirical tests with 212 participants and found that personal factors and behavioral intention were decisive factors affecting users’ willingness to adopt smart wearable devices, influenced by the wearable technical functions.

In addressing research question three (RQ3), this study identified five significant types of perceived outcomes (contributions) of the DWPs in the acceptance model, including (a) health and fitness, (b) enjoyment outcome, (c) social value, (d) biomimicry application, and (e) market growth. The most commonly reported perceived advantage was health and fitness outcomes, referring to the DWPs’ abilities in enhancing and monitoring users’ health and fitness, aligning with the primary (five) functionalities of the DWPs, including smartwatches, fitness trackers, medical devices, and robotics. Previous research confirms that health monitoring features, including heart rate, sleep, and physical activity tracking, enhance user motivation and intention for sustained usage, thus contributing to general health [103]. Muzny et al. [20] and Lu et al. [99] highlighted that wearable health technologies facilitate behavioral modification and preventative healthcare, rendering them especially attractive to health-conscious consumers.

This review revealed that the DWPs were not limited to health outcomes; they contribute significantly to promoting users’ enjoyment through hedonic pleasure and experiential engagement, as well as contributing to users’ social values through enhancing users’ social recognition and socialization. Hedonic pleasure, defined as the pursuit of fun, excitement, and pleasure, was increasingly critical in understanding consumer behavior in digital consumption. Numerous studies have underscored the significance of perceived enjoyment and pleasure in the user adoption of wearable devices. Wu et al. [69] discovered that enjoyment, as a hedonic motive, strongly affects users’ intentions to utilize smartwatches, especially among customers attuned to fashion and technology. Furthermore, DWPs designed with PU and appearance functions increasingly function as social tools that fulfill users’ aspirations for connection, recognition, and belonging. Devices like sport wearables, smartwatches, and fitness trackers enable users to share performance achievements, engage in virtual communities, and exhibit technological proficiency, thus augmenting social identity and contributing to overall social values. Prior research confirmed that social visibility and social value substantially affect the adoption of wearable devices, particularly among younger, trend-oriented users [12,65] and contribute significantly to the biomimicry application by promoting bio-inspired technology and self-healing. DWPs designed with PU, PEOU, and biomimetic functions significantly contribute to biomimicry as a whole. More specifically, studies in biomimicry confirmed that recent biomimetic designs in DWPs drawing inspiration from biological systems have improved key product attributes, such as sensor sensitivity, thermal management of the device, and bio-inspired design, that directly influence user satisfaction and adoption intentions, thus contributing to the biomimicry and consumer acceptance field [8,16,26].

Recently, digital wearables, such as smart medical devices, sensors, and robotics, have contributed significantly to the expansion and acceleration of market growth, especially for the wearables that provided the functions of PEOU, aesthetic design, biomimetic features, a high level of privacy and security. These functions constituted the fundamental value propositions that stimulated customers’ interest and ensured ongoing adoption. As customer expectations shift towards more functional, stylish, and safe devices, DWPs are becoming increasingly appealing to a broader audience, therefore enhancing industry competitiveness and economic growth [1,4,75]. Kim and Chiu [1] reported that technical features such as ease of use significantly influence purchasing intention and increase market demand towards these wearables. Similarly, Chang and Lin [89] highlighted that as wearable robotics incorporate more advanced features, such as biometric sensors and bio-inspired design, they would experience broader adoption across various sectors, including healthcare and fashion industrial applications.

This systematic literature review highlighted the distinctive technical functions of DWPs in the consumer acceptance model moderated by consumer behavior. The thematic analysis showed the comprehensiveness of the digital wearables market by providing a variety of fitness, health, and beauty wearables, sensors, and robotics with various technical functions designed to promote users’ health and fitness and social and engagement values and contribute to biomimicry and market growth in a broader sense. This increased interest in the wearables market was driven by consumer acceptance and behaviors, where 57.90% of the reviewed empirical studies demonstrated that the emergent technical functions of DWPs significantly influence consumer acceptance of these products. Moreover, the findings clarify how biomimetic features can influence user acceptance and perceived outcomes. For instance, self-healing materials or skin-mimicking structures may enhance users’ trust, intention, and acceptance by promoting safety and economic attributes and optimizing the wearable usage. This connection underscores how biomimetic innovations not only improve technical performance but also shape user attitudes and intentions towards the DWPs market. Therefore, integrating biomimetic principles into the traditional technical functions (PU and PEOU) would increase the consumer adaptation and acceptance of the DWPs.

### 5.1. Theoretical and Practical Implications of the Study

Despite growing academic interest in wearable technologies, existing studies remain fragmented, typically examining technical functions and consumer behavioral factors in isolation. This fragmentation limits the development of integrated acceptance models that explain how advanced features, particularly bio-mimetic innovations, interact with user perceptions to shape adoption decisions. Addressing this gap is critical for building comprehensive frameworks that can enhance both theoretical modeling and practical design strategies in the DWPs sector.

Therefore, from a theoretical perspective, the findings addressed gaps in the literature by enhancing existing acceptance models, specifically TAM, through the inclusion of biomimetic innovation and aesthetic design as critical constructs alongside PU, ease of use, and privacy and security. It expands the understanding of previous studies that mainly focus on the technical aspects (e.g., sensor design or development, and functional performance) by providing an inclusive inter-disciplinary perspective that integrates technical functions of DWPs with user-centered outcomes such as user behavior and acceptance impact. The study establishes a multi-dimensional consumer acceptance model for DWPs, which captures the interplay between technical functionalities and moderating behavioral factors such as user attitudes, social influence, and demographic variables. In general, there is a lack of evidence on a comprehensive typology framework for DWPs. Existing studies often classified wearable products based on specific aspects, for example, by function (especially in health monitoring or fitness) or user context (medical or consumer); however, these remain fragmented based on discipline-specific [98,102]. The current study sought to fill this gap in the existing literature by enhancing the comprehensive understanding of the different types and technical functions of DWPs and their various outcomes. It provided eight different types of digital products among health, robotics, sport and fitness, and fashion wearables.

Additionally, the study expanded the knowledge of TAM by integrating a bio-inspired design and bionic features such as self-healing materials and skin-mimicking structures into the acceptance model. This research adds a novel biomimetic dimension to the field of consumer technology adoption. Besides, dynamic AI integration was a core driver of PU in bionic wearables. Therefore, the current review also contributes directly to biomimetics by integrating the biomimetic innovation indicators into the other technical functions of DWPs in the TAM.

From a practical implication, the study revealed the different types of wearable products from various contexts, identifying their various technical functions that contribute to the users and the DWP industry by enhancing productivity and improving overall operational efficiency, thereby contributing to the users’ overall satisfaction. The four recognized technical functions, wearable technology, design, biomimicry, and privacy, must be regarded as strategic design criteria to improve customer involvement, satisfaction, and market competitiveness. Thus, the study provided critical insights for designers, engineers, manufacturers, and marketers of wearable devices. Developers might gain advantages from user-centered and bionic designs incorporated with intelligent assistive technologies that enhance wearability, comfort, health, and fitness for wearable technology users. DWPs users also benefit from the provided technical functions based on the typologies for better understanding and identifying the proper types for their lifestyle and needs. Consequently, the results of this paper provide a conceptual framework for developers and researchers to comprehend the technological functionalities of different DWPs that enhance user engagement in the digital wearables market and industry.

### 5.2. Limitations and Future Directions

The limitations of this systematic review were as follows: first, the comprehensiveness of the scope of the reviewed topic, particularly in studying the biomimicry in consumer acceptance. Despite the significant findings on biomimetic technology, the biomimetic approach was investigated in only 15.78% of the selected studies. This percentage reflected the scarcity of studies on the biomimicry and bionic functions in consumer acceptance. Second, all journal articles included in the current review were authored in English and published within the past decade. Consequently, articles written in languages other than English and published before 2014 were omitted from the analyses. Third, this systematic review has an exclusive inclusion of peer-reviewed journal articles, which may lead to publication limitation by omitting gray literature and industry reports. These sources may offer additional perspectives on practical applications, emerging technologies, or implementation challenges. However, the current systematic review applies a rigorous methodology by including only quality studies selected through the NOS checklist of Wells et al. [44] and the PRISMA framework of Moher et al. [40]. Fourth, there are also limitations in the sampling of the selected studies. This review focused on individual consumer behavior in general. Addressing this limitation in future reviews could enhance the comprehensiveness of evidence synthesis in this field.

While this review offers a comprehensive synthesis of current knowledge on DWPs and their technical functions in consumer acceptance, future research could empirically investigate user perceptions of emerging technologies such as biomimetic innovation through qualitative, quantitative, or mixed-method methods. Besides, clinical adoption reflects a significant use case for bionic and intelligent assistive technologies and was not deeply examined in this review. Therefore, future studies should develop a comprehensive conceptual framework extending the existing TAM to account for the bionic compatibility, intelligent assistive technology, and bio-sensor fusion offered by wearable robotics while addressing ethical, security, and privacy concerns. Such work would help uncover nuanced adoption barriers and value perceptions associated with advanced features and could improve explanatory power. Additionally, cross-cultural comparative studies could explore contextual differences in consumer intentions, further enriching theoretical models and providing targeted guidance for designers and marketers in DWPs’ markets. Furthermore, there is a critical need for further comprehensive empirical clinical research that studies digital wearable robotics and artificial intelligence technologies in the user adaptation model.

## 6. Conclusions

This systematic review provided an overview of recently published studies on smart wearables in the consumer acceptance model. A comprehensive systematic review, including bibliometric, thematic, and descriptive analyses, was conducted to tackle three principal research concerns, including types of DWPs, their technical functions, and related outcomes in the consumer acceptance model. This systematic analysis highlights the increasing importance of DWPs in consumer markets, demonstrating their expanding incorporation into healthcare, fitness, fashion, and daily life. A comprehensive study of 38 peer-reviewed research identified eight primary categories of DWPs, including smartwatches and medical robotics, and illustrated that their technical functionalities significantly impact customer acceptability.

The investigation indicated that four fundamental technical functions, PU and ease of use, attractive design, biomimetic innovation, and security and privacy, consistently forecast customer acceptance. Biomimetic attributes, including self-healing structures, skin-replicating materials, and thermal regulation, markedly improve user comfort, trust, and sustained engagement. The integration of wearable technology and AI with fashion and user-centered design signifies a transition towards more personalized and lifestyle-focused products. This consumer acceptance model was also discovered to be influenced by consumer behavior, encompassing personal, social, and behavioral attitudes, or behavioral intentions, highlighting the need for designers and developers to account for the wider social and psychological settings of consumers. The perceived outcomes of these DWPs encompassed health and fitness, enjoyment, social value, biomimicry applications, and market expansion.

In conclusion, the findings reinforce the value of a biomimetic and user-centric design incorporated with bionic and AI technology and a traditional functional approach in the development of wearable robotic technologies. Such technologies would be essential for enhancing consumer acceptance and fostering sustainable growth in the wearable technology industry.

## Figures and Tables

**Table 1:** The search strategy keywords used in the selected three electronic databases.

Variable 1: Exposure		Variable 2: Outcome		Variable 3: Moderator
(“digital wearable products” OR “smart wearable technology” OR “wearable devices” OR wearables OR “wearable technology” OR “smartwatches” OR “fitness trackers” OR “trackers” OR “smart glasses” OR “clothing” OR “rings” OR “jewelry” OR sensor) AND/OR (“technical functions” OR “functional features” OR “technical capabilities” OR “perceived usefulness” OR “ease of use” OR factors OR functions)	AND	(intention OR acceptance OR adoption OR perception)	AND/OR	(behavior OR behaviour* OR attitude OR social factor OR personal factor)

**Table 2:** Sources with the most publications.

Journals	Publisher	Years	No.
Sensors	MDPI	2014	1
Technology in Society	Elsevier	2016	3
Computers in Human Behavior	Elsevier	2016	3
Computer Methods and Programs in Biomedicine	Elsevier	2018	1
International Journal of Human–Computer Interaction	Taylor & Francis	2019, 2021	2
Digital Health	Sage	2020, 2022	2
Electronic Markets	Springer	2021	1
npj Digital Medicine	Nature	2021, 2023	2
Human–Computer Interaction	Taylor & Francis	2023	1
International Journal of Environmental Research and Public Health	MDPI	2023	1
Frontiers in Public Health	Frontiers	2024	1

**Table 3:** Summary of the keyword from the co-occurrence analysis.

Theme	Keyword/Term	Occurrence (Link)
DWPs and their functions	smartwatch	10 (53)
	wearable technology	7 (44)
	wearable device	7 (43)
	wearables	6 (33)
	data privacy	3 (24)
	fashion	3 (21)
	design	3 (16)
	biomimetic	2 (15)
	data security	2 (18)
	artificial intelligence	2 (13)
	self-healing	2 (13)
	and tracking	2 (13)
	visual aesthetic	2 (11)
	medical sensor	2 (10)
	trackers	2 (10)
	biomimetic structure	1 (10)
	self-healing materials	1 (10)
	accelerometer	1 (10)
	health management	1 (10)
Consumer acceptance	continuance intention	4 (22)
	intention	3 (19)
	TAM	3 (19)
Users’ behavior	behavior	4 (22)
	social influence	3 (19)
	attitude of use	3 (19)
Perceived outcomes	health	3 (22)
	perceived enjoyment	2 (18)
	biomimetic	2 (15)
	health outcomes	2 (14)
	self-healing	2 (13)
	socialization	1 (12)
	social media	1 (12)
	social comparison	1 (10)

**Table 4:** Types of DWPs and the consumer acceptance model.

Type of DWPs	N (%)	TF Category	TF Code	CA	UB	Source
Wristwatch accelerometer	1 (2.63%)	Wearable Technology (PU)	Health monitoring	Yes	No	[50]
Smartwatch	20 (52.26%)	Wearable Technology (PU)	Health monitoring	No	Yes (PF, SF)	[46,65]
			Fitness tracking	Yes	Yes (PF, SF, AoU, BI)	[48,54,64,65,68]
			Productivity	Yes	Yes (PF, AoU, BI) *	[61]
			Lifestyle monitoring	Yes	Yes (SF)	[47,55,63,66]
			Data feedback	Yes	Yes (PF, SF, AoU, BI)	[46,48,64]
		Wearable Technology (PEOU)	Ease of use	Yes	Yes (PF, BI)	[52,59,60,67,68,69]
		Appearance & Design	Visual appeal	Yes	Yes (PF, SF)	[51,55,70]
			Fashion fusion	Yes	Yes (PF, SF)	[55,70]
			UI aesthetics	Yes	Yes (PF)	[71]
		Security & Privacy	Data encryption	Yes	Yes (PF)	[74]
			Identity authentication	Yes	Yes (PF)	
			Anonymity protection	Yes	Yes (PF)	[74]
			Privacy policy	Yes	Yes (PF)	
Smart medical devices and robotics	8 (21.05%)	Wearable Technology (PU)	Health monitoring	Yes	Yes (PF)	[30,49,58]
			Data feedback	Yes	Yes (PF)	[30]
			AI	Yes	No	[49]
		Wearable Technology (PEOU)	Wearing comfort	Yes	No	[31,77]
		Security & Privacy	Data encryption	Yes	Yes (PF)	[76]
			Identity authentication	Yes	Yes (PF)	[76]
			Anonymity protection	Yes	Yes (PF)	[76]
			Privacy policy	Yes	Yes (PF)	[76]
		Biomimetic Innovation	Self-healing material	Yes	No	[77]
			Skin-mimicking structure	No	No	[28,31]
			Thermal regulation	Yes	No	[77,78]
			biomimetic structure	No	No	[28]
Smart glass (google glass)	2 (5.26%)	Wearable Technology (PEOU)	Ease of use	Yes	Yes (SF, BI)	[62,67]
Sports wearables	1 (2.63%)	Wearable Technology (PU)	Fitness tracking	Yes	No	[1]
		Wearable Technology (PEOU)	Ease of use	Yes	No	
Wearable fashions (clothing & jewelry)	4 (10.52%)	Wearable Technology (PU)	Lifestyle monitoring	No	No	[66]
			AI	Yes	Yes (AoU)	[57]
		Wearable Technology (PEOU)	Ease of use	Yes	Yes (PF)	[69]
		Appearance & Design Aesthetics	Fashion fusion	Yes	Yes (BI)	[89]
		Biomimetic Innovation	Skin-mimicking structure	Yes	Yes (BI)	[89]
			biomimetic structure	No	Yes (AoU)	[57]
Wearable fitness devices	7 (18.42%)	Wearable Technology (PU)	Fitness tracking	Yes	Yes (BI)	[48,68]
			Lifestyle monitoring	No	No	[66]
			Data feedback	No	Yes (BI)	[48]
		Wearable Technology (PEOU)	Ease of use	Yes	No	[67,68]
		Appearance & Design Aesthetics	Visual appeal	No	No	[73]
		Security and Privacy	Data encryption	Yes	Yes (PF)	[74,76]
			Identity authentication	Yes	Yes (PF)	[76]
			Anonymity protection	Yes	Yes (PF)	[74,76]
			Privacy policy	Yes	Yes (PF)	[76]
General wearable technology	4 (10.52%)	Wearable Technology (PU)	Health monitoring	Yes	Yes (PF, AoU, BI)	[53,56]
		Appearance & Design Aesthetics	Visual appeal	Yes	Yes (AoU)	[56,72]
		Security and Privacy	Privacy policy	No	No	[75]

* Note: TF: technical functions; CA: consumer acceptance; UB: user behavior; PF: personal factor, SF: social factor, AoU: attitude of use, BI: behavioral intention.

**Table 5:** Technical functions of DWPs and the consumer acceptance model.

No	Category		Codes	N (%)	CA *	User Behavior				Source
						PF *	SF *	AoU *	BI *	
1	Wearable Technology	Perceived Usefulness (PU)	(a) Health monitoring	7 (18.42%)	√	√		√	√	[30,46,49,50,53,58,65]
			(b) Fitness tracking	6 (15.78%)	√	√	√	√	√	[1,48,54,64,65,68]
			(c) Enhance productivity	1 (2.63%)	√	√		√	√	[61]
			(d) Lifestyle monitoring	5 (13.15%)	√			√		[47,55,56,63,66]
			(f) Data feedback	4 (10.52%)	√	√		√	√	[30,46,48,64]
			(e) AI	2 (5.26%)	√			√		[49,57]
		Perceived Ease of Use (PEOU)	(a) Interface Simplicity	1 (2.63%)	√	√				[60]
			(b) Ease of Use	8 (21.05%)	√	√	√		√	[1,52,59,60,62,67,68,69]
			(c) Wearing Comfort	2 (5.26%)	√					[31,77]
2	Appearance & Design		(a) Visual Appeal	6 (15.78%)	√	√	√	√		[51,55,56,70,72,73]
			(c) Fashion Fusion	3 (7.89%)	√	√	√		√	[55,70,89]
			(d) UI aesthetics	1 (2.63%)	√	√				[71]
4	Biomimetic Innovation		(a) Self-healing structure	2 (5.26%)	√					[28,77]
			(b) Skin-mimicking structure	3 (7.89%)	√				√	[28,31,89]
			(c) Thermal regulation structure	2 (5.26%)	√					[77,78]
			(d) Biomimetic structure	2 (5.26%)				√		[28,57]
3	Security and Privacy		(a) Data Encryption	2 (5.26%)	√	√				[74,76]
			(b) Identity Authentication	1 (2.63%)	√	√				[76]
			(c) Anonymity Protection	2 (5.26%)	√	√				[74,76]
			(d) Privacy Policy	2 (5.26%)	√	√				[75,76]
5	User Behavior		(a) PF *	10 (26.31%)	√	/	/	/	/	[30,46,51,53,54,60,61,69,70,71,76]
			(b) SF *	5 (13.15%)	√	/	/	/	/	[55,62,64,65,70]
			(c) AoU *	5 (13.15%)	√	/	/	/	/	[53,56,57,61,64]
			(d) BI *	6 (15.78%)	√	/	/	/	/	[48,53,61,62,89]

* Note: TF: technical functions; CA: consumer acceptance; UB: user behavior; PF: personal factor, SF: social factor, AoU: attitude of use, BI: behavioral intention.

**Table 6:** Perceived outcomes and related DWPs in the consumer acceptance model.

No	Perceived Outcomes	Codes	No (%)	Wearable Technology: PU	Wearable Technology: PEOU	Appearance & Design	Biomimetic Innovation	Security & Privacy	Acceptance	Source
1	Health and Fitness	Health motivation	23 (60.52%)	Yes	Yes	Yes	Yes	Yes	Yes	[1,28,30,31,46,47,48,49,52,53,59,60,61,65,66,67,68,69,70,71,74,76,89]
		Fitness performance		Yes	Yes	Yes	Yes	Yes	Yes	
2	Enjoyment	Hedonic pleasure	8 (21.05%)	Yes	No	Yes	Yes	No	Yes	[54,56,61,64,70,71,78,89]
		Perceived enjoyment		Yes	No	Yes	No	No	Yes	
3	Social value	Social recognition	5 (13.15%)	Yes	No	Yes	No	No	Yes	[30,64,65,70,71]
		Socialization		Yes	No	Yes	No	No	Yes	
4	Biomimicry application	Bio-inspired technology	5 (13.15%)	Yes	Yes	No	Yes	No	Yes	[28,31,77,78,89]
		Self-healing		Yes	Yes	No	Yes	No	Yes	
5	Market growth		4 (10.52%)	No	Yes	Yes	Yes	Yes	Yes	[62,72,75,89]

## Data Availability

The data presented in this study are openly available in Zenodo at 10.5281/zenodo.11054419. All of the selected study data are available in Appendix A and Supplementary Information.

## Supplementary Materials

The following supporting information can be downloaded at https://www.mdpi.com/article/10.3390/biomimetics10080483/s1: Table S1: Main summary and data extraction table of the selected studies; Supplementary Information S2: Summary data extraction and synthesis; Table S3: The PRISMA checklist; Table S4: Quality assessment using NOS.

## Abbreviations

The following abbreviations are used in this manuscript:

AI	artificial intelligence
BTI	biomimetic technological innovation
DWPs	digital wearable products
IoT	Internet of Things
PEOU	perceived ease of use
PU	perceived usefulness
TAM	technology acceptance model

## Appendix A

**Table A1:** Summary of the selected studies.

No.	Source	Year	Country	Journal	Wearable Type	Objective	Methodology				Finding
							Methods and Validation	Variables/Themes	Framework/Theory	Analyzing Tool/Test	
1.	Garcia-Ceja et al. [50]	2014	Mexico	Sensors (Switzerland)	Wristwatch accelerometer	To recognize long-term activities using wristwatch accelerometer data	**Method:** Quantitative (Experimental approach) **Sample size:** N = 21 days of data from 2 subjects **Validation:** Construct validation, Internal validation	**IV:** Acceleration data from wristwatch *(performance and use);* **DV:** Long-term activities (shopping, commuting, working, etc.)	Hidden Markov Models (HMM), Conditional Random Fields (CRF)	Viterbi algorithm, k-minimum-consecutive-states constraint	They have used accelerometers from smartphones to classify sporting activities. HMMs and CRFs were used to perform the segmentation. It was shown how adding additional information to the models helped to increase the overall accuracy of the tested approaches.
2.	Yang Y. et al. [77]	2015	China	Nano Energy	Smart medical device (Motion sensors)	To develop a flexible, self-healing motion sensor using nanocomposites.	**Method:** Quantitative (Experimental approach) **Sample size:** N = 5 subjects **Validation:** Construct validation, Internal validation	**IV:** Use of recoverable motion sensor *(acceptance)* **DV:** Dielectric permittivity, self-healing efficiency, effectiveness, heat regulation	Self-healing materials, Percolation theory	Dielectric property measurements, Mechanical testing	With the incorporation of surface-modified CCTO nanoparticles, the hybrid film offers an improved dielectric property and capacitance retention based on the DA reaction. The sensor shows good sensitivity and recovery of property based on the self-healing polymer matrix.
3.	Jung, Y. et al. [51]	2016	South Korea	Computers in Human Behavior	Smartwatch	To understand potential consumers’ perceptions of smartwatches	**Method:** Quantitative (Experimental approaches & Conjoint survey) **Sample size:** N = 123 respondents **Validation:** Content validation, Construct validation	**IV:** Smartwatch attributes (PU, PEOU, brand, display shape (design), etc.) **DV:** Consumers’ perceptions and attitude *(acceptance)* **PV:** Personal Factor (age, gender, time spent using smartphones)*: (user behavior)*	NA	Part-worths, Kendall’s tau, relative importance, ANOVA	Display shape and standalone communication are critical factors. Yet, they suggested that an overemphasis on design elements and an underestimation of the importance of functionality can lead to late diffusion or failure of wearables.
4.	Wu, L. H. et al. [60]	2016	Taiwan	Computers in Human Behavior	Smartwatch	To propose a research model for smartwatch context and identify potential consumers	**Method:** Quantitative (Online questionnaire survey) **Validation:** Construct validation, internal consistency	**IV:** Perceived relative advantage, perceived ease of use (PEOU), perceived compatibility, perceived result demonstrability, perceived enjoyment, perceived social influence, behavioral intention **DV:** Intention to use the smartwatch *(acceptance)* **MV:** Personal Factor (age and gender): *(user behavior)*	Unified Theory of Acceptance and Use of Technology (UTAUT), Innovation Diffusion Theory (IDT), Technology Acceptance Model (TAM)	PLS-SEM	The proposed model fits the smartwatch context well, and perceived relative advantage significantly affects behavioral intention.
5.	Choi and Kim [61]	2016	South Korea	Computers in Human Behavior journal	Smartwatch	To examine whether factors related to fashion products affect the intention to use smartwatches	**Method:** Quantitative (Online questionnaire survey) **Sample size:** N = 562 **Validation: Construct validation, internal consistency**	**IV:** Perceived usefulness (PU), PEOU, perceived enjoyment, perceived self-expressiveness, vanity, need for uniqueness **DV:** Behavioral Intention (user behavior) & Intention to use *(acceptance)* **MV:** Attitude of use and personal factor *(user behavior)*	Technology Acceptance Model (TAM)	PLS-SEM	Fashion-related factors significantly influence the intention to use smartwatches. A significant positive relationship was found between attitude and behavioral intention to use smartwatches. The direct effect of PU on attitude towards smartwatch usage was statistically significant, while PEOU showed a negligible direct effect on the attitude.
6.	Li, X. et al. [30]	2017	USA	PLoS Biology	Smart medical devices (Scanadu Scout, iHealth-finger, etc.)	To investigate the use of wearable biosensors for monitoring health and detecting diseases	**Method:** Quantitative (Observational) **Sample size:** N = 43 participants **Validation:** Construct validation, Internal validation	**IV:** Device criteria for selection, physiological parameters (HR, SpO2, skin temperature) **DV:** Activity & health status, personal norms, or intention to use the DWPs *(acceptance)* **MV:** differences in individuals *(personal factor: user behavior)*	NA	Bland-Altman method, Pearson correlation, peak detection	It indicated that the information provided by wearable sensors is physiologically meaningful and actionable. Developed a computational algorithm for personalized disease detection using such sensors
7.	Chun, J. et al. [52]	2018	USA	Human Factors and Ergonomics in Manufacturing	Smartwatch	To explore usability issues of smartwatches and suggest guidelines for improved operation.	**Method:** Qualitative (diary study and task performance) **Sample size:** N = 30 participant **Validation:** Construct validation; Content validation; Internal validation	**IV:** PEOU **DV:** Usability ratings, Task performance, preference (satisfaction and intention: *acceptance*)	Usability principles (Information Display, Control, Learnability, Interoperability, Preference)	Qualitative analysis, Task performance tests	The study found that smartwatches were predominantly used for quick information checks. Participants effectively used their smartwatches when multitasking, especially when their hands were occupied with a concurrent task. Participants had a clear preference for which smart device to use for a given task.
8.	Nunes and Arruda Filh [62]	2018	Brazil	Innovation and Management Review	Smart Google Glass	To analyze consumer behavior in relation to Google Glass	**Method:** Qualitative (ethnography via passive observation) **Sample size:** N = 86 (unique posts) **Validation:** Content validation	**Unit of analysis:** Socially satisfied, socially constrained, early adopters, ease of use, *social factor, attitude, behavioral intention (consumer behavior)*, intentions *(consumer acceptance)*	Diffusion of Innovations Theory, Technological Convergence, Utilitarianism, Hedonism	Thematic Content Analysis	The results showed that Google Glass faced a series of problems related to the adoption and diffusion of the innovation.
9.	Kekade et al. [53]	2018	Global (Europe, America, Asia, and Australia)	Computer Methods and Programs in Biomedicine	General: Wearable devices (WDs)	To determine the usefulness and actual use of wearable devices among the elderly population.	**Method:** Mixed Method (Systematic review and survey) **Sample size:** SR: 31 studies; Survey: 233 respondents **Validation:** Construct validation; Content validation	**IV:** PDWs for health management **DV:** Use of wearable devices, Willingness to pay *(attitude of use and behavioral intention: user behavior)* & Intention to use *(acceptance)* **PV:** age, gender, health status, living with, current residence *(personal factors: user behavior)*	NA	Qualitative synthesis, Survey analysis	More than 60% of elderly people were interested in the future use of wearable devices, and preferred future use to improve physical and mental activities. Wearable devices can benefit the elderly population, but awareness and usage are limited.
10.	Kheirkhahan et al. [46]	2019	USA	Journal of Biomedical Informatics	Smartwatch	To develop a smartwatch-based framework for real-time and online assessment and mobility monitoring (ROAMM).	**Method:** Quantitative (Experimental testing) **Sample size:** N = 5 participants **Validation:** Construct validation; Internal validation	**IV:** Sensor data (accelerometer, gyroscope, heart rate: PU) **DV:** Mobility and activity data **MV:** Health status *(personal factor: user behaviour)*	NA	Smartwatch application, Server software, Data visualization	The ROAMM framework allows for real-time monitoring of physical activity and health events. Recalled changes in pain explained only 15% of the variance in momentary changes in pain.
11.	Kim and Chiu [1]	2019	South Korea	International Journal of Sports Marketing and Sponsorship	Sports wearables	To investigate consumers’ acceptance and use of sports and fitness wearable devices based on technology readiness	**Method:** Quantitative (Mall-intercept personal survey) **Sample size:** N = 247 **Validation:** Construct validation, internal consistency	**IV:** Positive technology readiness (PTR), negative technology readiness (NTR) **Mediators:** PEOU and PU **DV:** Intention to use sports wearables *(acceptance)*	Technology Readiness and Acceptance Model (TRAM)	PLS-SEM	Positive TR significantly influences PEOU and PU, which in turn affect the intention to use sports wearables.
12	Weiss et al. [47]	2019	USA	IEEE Access	Smartwatch	To evaluate the feasibility of using smartphone and smartwatch sensors for biometric authentication and identification based on daily activities.	**Method:** Quantitative (Experimental study) **Sample size:** N = 51 participants **Validation:** Construct validation; Content validation; Internal validation	**IV:** Sensor data **DV:** Authentication accuracy, Identification accuracy	Behavioral biometrics	k-Neighbors, Decision Tree, Random Forest	The results showed that motion-based biometrics using activities of daily living is feasible using a commercially available. The performance for the authentication task improves rapidly as more training data is added, and the improvement continues when the maximum of 170 s of data per activity is reached.
13.	Tan et al. [78]	2020	China	Nature Communications	Smart medical device (Strain sensors)	To develop a wearable strain sensor with enhanced thermal management.	**Method:** Quantitative (Experimental approach) **Sample size:** N = 3 **Validation:** Construct validation, External validation	**IV:** Strain sensor design **DV:** Strain, Temperature, Thermal conductivity	Thermal management, Electromechanical performance	Thermal conductivity measurements, Cytotoxicity tests	The as-casted TPU-BNNS film leads to enhanced thermal conductivity, helping rapid heat transmission to the environment, while the porous electrospun fibrous membrane layer results in thermal insulation, functioning as a skin protector. The sensor demonstrates excellent thermal stability and biocompatibility.
14.	Chang and Lin [89]	2020	Taiwan	Interaction Studies. Social Behaviour and Communication in Biological and Artificial Systems	Wearable Fashion Products	To explore narrative design and cultural semantics in wearable and fashionable interaction design	**Method:** Qualitative (Case study and interview) **Sample size:** N = 6 **Validation:** Construct validation, Content validation, External validation	**Unit of analysis:** technological function: fashionable interaction design, design aesthetic, nature as a model (biomimetic); human cognition *(behavioral intention)*: intention and *(acceptance)*	Design anthropology	Thematic analysis for principles of design anthropology	The current design philosophy and practice in Taiwan is directed toward producing products with cultural significance to compete in the global market. The main reason for the abandonment of wearable devices is that many consumers believe that the function and options are still limited. The cultural semantics and design enhance product value and user experience.
15.	Bolen [63]	2020	Turkey	Technology in Society journal	Smartwatch	To examine factors affecting traditional wristwatch users’ intentions to switch to smartwatches	**Method:** Quantitative (Online questionnaire survey) **Sample size:** N = 234 **Validation:** Construct validation, internal consistency	**IVs:** Relative advantage, complexity, perceived product lifetime, procedural switching costs, financial switching costs **DV:** Switching intention *(acceptance)*	Diffusion of Innovations Theory (DIT)	PLS-SEM	Relative advantage positively affects switching intention, while financial switching costs have a negative impact. It showed that the impact of perceived product lifetime on switching intention is seen only indirectly through financial switching costs.
16.	Lee [70]	2020	USA	Journal of Consumer Behaviour	Smartwatch	To examine how visual typicality affects consumer adoption of wearables through psychological antecedents	**Method:** Quantitative (Online questionnaire survey) **Sample size:** N = 409 **Validation:** Construct validation, Content validation, External validation	**IV:** Visual Typicality **DV:** Purchase Intention *(acceptance)* **MV:** Effort Expectancy Performance Expectancy, Playfulness, Social Influence, Gender *(personal factor and social factor: user behavior)*	Technology Acceptance Model (TAM), Unified Theory of Acceptance and Use of Technology (UTAUT)	Structural Equation Modeling (SEM)	Visual typicality negatively impacts perceived performance and playfulness, leading to lower purchase intention.
17.	Lewis et al. [48]	2020	USA	Digital Health	Wearable fitness devices (e.g., Fitbit, Apple smartwatch)	To explore which features of wearable fitness trackers are used and deemed helpful	**Method:** Mixed Method (Online survey and focus group discussions) **Sample size:** Survey: N = 47 (wearable owners), Focus groups: N = 7 **Validation:** Construct validation	**IV:** Wearable features **DV:** Helpfulness ratings, users’ intention behavior *(user behaviour)*	NA	Descriptive statistics, Thematic analysis	Motivational cues, general health information, and challenges are the most significant features for the users’ intentions.
18.	Cillier [74]	2020	South Africa	Health Information Management Journal	Wearable fitness devices (e.g., fitness trackers) and Smartwatch	To investigate privacy and information security issues associated with wearable health devices	**Method:** Quantitative (Cross-sectional, online questionnaire survey) **Sample size:** N = 106 **Validation:** Construct Validation	**IV:** Use of wearable health devices **DV:** Privacy awareness, Information security knowledge	NA	Descriptive statistics (SPSS Version 25)	Users were lacking awareness of privacy risks and information security issues related to wearable devices.
19.	Mahadevan et al. [49]	2021	USA	npj Digital Medicine	Smart medical device (Wrist-worn devices e.g., GeneActiv)	To develop and validate digital measures for nighttime scratch and sleep using wrist-worn devices	**Method:** Quantitative (Experimental approach) **Sample size:** N = 33 (participants) **Validation:** Construct validation	**IV:** Accelerometer data **DV:** Nighttime scratch and sleep measures	NA	Python, MATLAB, SHAP analysis	Observed a weak correlation between scratch endpoints and TST. These results indicate that increased nighttime scratching does not necessarily contribute to shorter sleep duration but may result in more disturbed sleep. Focused on using accelerometer and temperature data sampled at 20 Hz to maximize battery life and believe these choices will improve patient compliance.
20.	Wang et al. [71]	2021	China	International Journal of Human-Computer Interaction	Smartwatch	To investigate the impact of symmetry, complexity, and screen shapes on user quality perceptions and continuous usage intention of smartwatches	**Method:** Quantitative (Experiment approach) **Sample size:** N = 200 **Validation:** Construct validation, Internal validation, External validation	**IV:** Symmetry, Complexity, Screen Shap**e** **DV:** Hedonic Quality, Pragmatic Quality, Continuous Usage Intention *(acceptance)* **MV:** *Personal factors (user behavior)*	NA	ANOVA, OLS Regression	They found that asymmetrical and complex designs induced higher hedonic quality; round screens also induced higher hedonic quality.
21.	Gupta et al. [64]	2021	India	Behaviour and Information Technology	Smartwatch	To investigate the continuance intention of smart fitness wearables by integrating expectation confirmation theory and social comparison theory.	**Method:** Quantitative (Questionnaire survey) **Sample size:** N = 684 **Validation:** Construct validation	**IV:** Perceived health outcomes, social comparison tendency **DV:** User satisfaction, *Continuance intention (acceptance)* **MV:** *Attitude of Use, Social Factor (user behavior)*	Expectation Confirmation Model (ECM), Social Comparison Theory	SEM	Perceived health outcomes and social comparison significantly influence user satisfaction and continuance intention. User satisfaction positively impacts continuance intention and intention to recommend.
22.	Siepmann, and Kowalczuk [54]	2021	Germany	Electronic Markets	Smartwatch	To investigate factors driving long-term smartwatch usage, focusing on emotional and health/fitness factors	**Method:** Quantitative (Online questionnaire survey) **Sample size:** N = 335 (smartwatch users) **Validation:** Construct validation	**IV:** Emotional factors Health/fitness factors **DV:** Continuance intention *(acceptance)* **CV:** *Personal factor: user behavior: (age, gender, etc)*	Expectation-Confirmation Model (ECM) extended with emotional and health/fitness factors	Covariance-based SEM	Emotional and health/fitness factors significantly impact smartwatch continuance intention. Satisfaction is a strong driver of continuance intention. The effect of age on continuance intention was positive and significant, indicating that older participants have a higher intention to use smartwatches continuously.
23.	Goyal et al. [31]	2022	USA	Sensors and Actuators A: Physical	Smart medical device (Wearable electrodes)	To develop a skin phantom that mimics the electrical properties of human skin for testing wearable electrodes.	**Method:** Quantitative (Experimental approach) **Sample size:** N = 5 **Validation:** Construct validation, Internal validation	**IV:** Porosity of the phantom’s upper layer **DV:** Impedance response, comfort, signal quality (ECG)	Biomimicry, Electrical impedance spectroscopy	Impedance spectroscopy, Bode plot analysis	The phantom accurately mimics human skin impedance and can simulate the impact of dry and hydrated skin on ECG signals.
24.	Basha et al. [55]	2022	Malaysia	Technology in Society	Smartwatch	To understand the factors that sustain long-term smartwatch usage, focusing on technology, fashion, and psychographic attributes	**Method:** Quantitative (Online questionnaire survey) **Sample size:** N = 275 (smartwatch users) **Validation:** Construct validation, External validation	**IV:** Technology-related features, Fashion-related features, Psychographic factors **DV:** Continuance intention *(acceptance)* **MV:** Social factor *(user behavior)*	Stimulus-Organism-Response (S–O-R) model and self-congruity theory	PLS-SEM, IPMA	Technology, fashion, and psychographic related attributes significantly influence smartwatch continuance intention, this could be influenced by the social aspects.
25.	Quach et al. [75]	2022	Australia	Journal of the Academy of Marketing Science	General: Wearable technology (social media, IoT, AI)	To understand the privacy tensions arising from firms’ use of digital technologies and their implications for firm performance	**Method:** Qualitative (Case study and interviews) **Sample size:** N = 15 (10 senior managers + 5 consumer informants) **Validation:** Construct Validation	**IV:** Data monetization, Data sharing **DV:** Firm performance, Privacy tensions	Structuration theory	Content analysis	Firms need to balance data monetization and sharing with privacy protection to maintain consumer trust and regulatory compliance.
26.	Lee [73]	2022	USA	Journal of Retailing and Consumer Services	Smart Fitness Trackers	To examine the impact of visual aesthetics on willingness-to-pay premium and the role of product category involvement	**Method:** Quantitative (Online questionnaire survey) **Sample size:** N = 423 **Validation:** Construct validation, Content validation, External validation	**IV:** Visual Aesthetics **DV:** Willingness-to-Pay (WTP) Premium **MV:** Product Category Involvement, Perceived Enjoyment	NA	Structural Equation Modeling (SEM)	Visual aesthetics positively impact WTP premium through perceived enjoyment; involvement moderates the effect.
27.	Wang et al. [65]	2022	China	Technology in Society	Smartwatch	To explore the factors affecting users’ continuance intentions for smart wearable products from a consumer-driven perspective.	**Method:** Quantitative (Online questionnaire survey) **Sample size:** N = 249 **Validation:** Construct validation	**IV:** Individual characteristics, e.g., utilitarian value (healthy routines, track daily activities), Social capital factors **DV:** Perceived value, Continuance intention *(acceptance)* **MV:** Social factor *(user behavior)*	Means-end chain theory	Structural equation modeling (SEM), Fuzzy-set Qualitative Comparative Analysis (fsQCA)	Face consciousness positively influences perceived value and continuance intention. Social capital and perceived value significantly affect continuance intention.
28.	Bakhshian and Lee [56]	2022	USA	International Journal of Human-Computer Interaction	General: Wearable technology (e.g., smart clothing and others)	To examine the effects of intrinsic and extrinsic attributes of wearables on consumer attitude and intention	**Method:** Quantitative (Online questionnaire survey) **Sample size:** N = 317 (college students) **Validation:** Construct validation	**IV:** Intrinsic attributes, Extrinsic attributes, PU, Visual Appeal **DV:** Intention of use *(acceptance)* **MV:** Consumer attitude of use *(user behavior)*	Functional-expressive-aesthetic (FEA) model, Technology Acceptance Model (TAM)	Confirmatory factor analysis, SEM	Tracking attributes significantly influence consumer attitude and intention to use wearables, attitude of use moderations the effect.
29.	Chen et al. [28]	2022	China	Biosensors	Smart medical device (wearable sensors and climbing robots)	To investigate matters related to different sensors on human skin by optimizing and fusing the two biomimetic self-adhesive structures.	**Method:** Quantitative (Experimental approach) **Sample size:** N = 2 adhesion biomimetics **Validation:** Method and results validation using Adhesion repeatability tests	**IV:** biomimetic materials and structures; **DV:** Sensing performance, flexibility, adhesion, self-adhesion, human health related issues, long-term monitoring	NA	Characterization analysis (characterization analysis and X-ray diffraction (XRD) analysis, Pulse waveform measurements, adhesion tests)	Both biomimetic adhesive structures of the sensors, designed with biomimetic characteristics, were functional in pulse wave tests and demonstrated good adhesion repeatability.
30.	Jeong and Choi [66]	2022	South Korea	SAGE Open	Smartwatch, Wearable fitness devices, Smart clothing	To identify factors influencing the purchase intention of wearable devices and examine the moderating role of consumers’ personal innovativeness.	**Method:** Quantitative (online questionnaire survey) **Sample size:** N = 512 **Validation:** Construct validation	**IV:** Wearability, social image, novelty, esthetics, relative advantage **DV:** Purchase intention *(acceptance)* **MV:** Personal innovativeness	Functional, Expressive, and Esthetic (FEA) consumer needs model	SEM	Social image, novelty, esthetics, and relative advantage significantly influence purchase intention. Personal innovativeness moderates the relationship between novelty and purchase intention.
31.	Rahman et al. [67]	2022	Bangladesh	Journal of Science and Technology Policy Management	Smartwatch, Wearable fitness devices, Google glasses	To investigate the factors driving teenagers’ behavioral intention to adopt wearable technologies and their intention to recommend others.	**Method:** Quantitative (Questionnaire survey) **Sample size:** N = 318 **Validation:** Construct validation	**IV:** Performance expectancy, Effort expectancy, Social influence, Facilitating conditions, price value, Hedonic motivation, habit **DV:** Intention to recommend *(acceptance)* **MV: B**ehavioral intention and attitude of use *(user behavior)*	Unified Theory of Acceptance and Use of Technology (UTAUT2), Theory of Planned Behavior (TPB)	SEM	Performance expectancy, social influence, facilitating conditions, and attitude significantly influence behavioral intention. Behavioral intention positively impacts intention to recommend.
32.	Hayat et al. [68]	2023	Malaysia	Digital Health	Wearable fitness devices, Smartwatch	To investigate the formation of intention to use wearable fitness devices (WFDs) and the role of health consciousness (HCS) and health motivation (HMT) in their adoption.	**Method:** Quantitative (Online questionnaire survey) **Sample size:** N = 525 **Validation:** Construct validation	**IV:** HCS, Perceived compatibility (PCM), Perceived product value (PPV), Perceived technology accuracy (PTA), PU **DV:** Intention to use WFDs, Use of WFDs *(acceptance)*	Health consciousness and health motivation theories	PLS-SEM	Perceived compatibility, product value, and technology accuracy significantly influence the intention to use WFDs. HMT significantly impacts the adoption of WFDs.
33.	Haseli et al. [57]	2023	Mexico	Technological Forecasting and Social Change	Wearable fashion products (jewelry)	To determine key evaluation criteria for smart jewelry selection for women and select the best alternative using BCM-MARCOS with fuzzy ZE-numbers.	**Method:** Qualitative (fuzzy numbers, fuzzy ZE-numbers, interviews with experts) **Sample size: N = 4 decision-makers, 6 experts** **Validation:** Construct validation	**IV:** Jewelry-related criteria, technology-related criteria, producer-related criteria **DV:** Attitude of use smart jewelry *(user behavior)*	Multi-Criteria Decision-Making (MCDM), Fuzzy number theory	compromise solution (MARCOS) methods using fuzzy ZE-numbers	Ringly Luxe Smart Bracelet was related to the women’s intention model and attitude of use as smart jewelry. Technology-related criteria were most prioritized,
34.	Johnson et al. [58]	2023	USA	npj Digital Medicine	Smart medical device: Wrist-worn activity monitor (ActiGraph Insight Watch, Ankle-worn activity monitor, Modus StepWatch)	To determine if mobile applications and wearable devices can quantify ALS disease progression through active and passive data collection.	**Method:** Quantitative (Longitudinal observational study) **Sample size: N = 40 participants** **Validation:** Internal validation, External validation	**IV:** Wearable device data **DV:** ALSFRS-R, ALSFRS-RSE, ROADS	NA	Linear Mixed Models (LMM), Correlation analysis	Wearable devices and smartphone data can effectively quantify ALS progression and may serve as novel outcome measures.
35.	Rapp [72]	2023	Italy	Human-Computer Interaction	General: Wearable technology	To propose a theoretical framework for understanding wearables as extensions of human intentionality and explore design implications	**Method:** Qualitative (diary study and interviews) **Sample size:** N = 64 (14 participants in diary study, 24 in user study, 30 in interview studies) **Validation:** Construct Validation	**IV:** Wearable design approach **DV:** User experience	NA		Internalistic design can enhance user experience by integrating wearables more closely with human perception and action.
36.	Thapa et al. [76]	2023	Australia	International Journal of Environmental Research and Public Health	Smart medical device (WIoMT and Biosensors)	To examine users’ perspectives of trust in the Wearable Internet of Medical Things (WIoMT) while also exploring the associated security risks.	**Method:** Quantitative (online, cross-sectional questionnaire survey) **Sample size:** N = 189 (aged 18 and over) **Validation:** Construct validation	**IV:** Intention to Use wearable medical things *(acceptance)* **DV:** Product factors, privacy risks, data security, personal data (user behavior)	Technology Acceptance Model (TAM)	Correlation analysis using IBM SPSS software and Microsoft Excel	Users intend to use digital devices based on trust factors related to security and privacy features.
37.	Wu and Lim [69]	2024	China	Frontiers in Public Health	Smartwatches, Smart clothing	To identify key factors influencing older adults’ willingness to adopt smart wearable devices and their impact mechanisms.	**Method:** Quantitative (Online questionnaire survey) **Sample size:** N = 389 **Validation:** Construct validation	**IV:** Performance expectancy, Effort expectancy, social influence, facilitating conditions, Hedonic motivation, price value, digital health literacy **DV:** Intention to technology, acceptance *(acceptance)* **Moderator:** Digital health literacy (*user behavior by PF*)	UTAUT2, Technology Readiness Index (TRI)	PLS-SEM	Performance expectancy, effort expectancy, social influence, facilitating conditions, hedonic motivation, and price value significantly affect older adults’ willingness to adopt smart wearable devices. Digital health literacy moderates the relationship between these factors and behavioral intention.
38.	Lu et al. [59]	2024	China	Telematics and Informatics Reports	Smartwatches (Apple Watch, Xiaomi Smart Band, Huawei Watch)	To explore the self-tracking practices of smartwatch users and the impact of technology and data on the body and self.	**Method:** Qualitative (Case study) **Sample size: N = 25 participants** **Validation:** Content validation, external validation	**IV:** Smartwatch usage patterns, data **DV:** Self-perception, satisfaction, and accepting the smartwatch *(acceptance)*	NA	Qualitative analysis, thematic coding	Smartwatches help in constructing technological bodies and enable digital care practices, enhancing users’ satisfaction and acceptance.
