# Supplementary material for: Technical Functions of Digital Wearable Products (DWPs) in the Consumer Acceptance Model: A Systematic Review and Bibliometric Analysis with a Biomimetic Perspective
Source: Biomimetics (Basel). 2025 Jul 22;10(8):483. doi: 10.3390/biomimetics10080483 (PMC12383983; doi:10.3390/biomimetics10080483)
Supplement: Supplementary file 1 [file biomimetics-10-00483-s001.zip › biomimetics-3722362-supplementary/Supplementary Document_S3_PRISMA_2025_checklist.pdf]

Supplementary Document 3

Table S3: PRISMA Checklist

Section and Topic	Item #	Checklist item	Location where item is reported (Page No)
**TITLE**			
Title	1	Technical Functions of Digital Wearable Products (DWPs) in the Consumer Acceptance Model: A Systematic Review with a Biomimetic Perspective.	Page 1
**ABSTRACT**			
Abstract	2	Design and use of wearable technology have grown exponentially, particularly in consumer products and service sectors, e.g., healthcare. However, there is a lack of a comprehensive understanding of wearable technology in consumer acceptance. This systematic review utilized a PRISMA on peer-reviewed articles published between 2014 and 2024 and collected on WoS, Scopus, and ScienceDirect. A total of 38 full-text articles were systematically reviewed and analyzed using bibliometric, thematic, and descriptive analysis to understand the technical functions of digital wearable products (DWPs) in consumer acceptance. The findings revealed five key functions: (i) wearable technology, (ii) appearance and design, (iii) biomimetic innovation, (iv) security and privacy, found in eight types of DWPs, among them smartwatches, medical robotics, fitness devices, and wearable fashions, significantly predicted the customers' acceptance moderated by the behavioral factors. The review also identified five key out-comes: health and fitness, enjoyment, social value, biomimicry, and market growth. The review proposed a comprehensive acceptance model that combines biomimetic principles and AI-driven features into the technical functions of the technical function model (TAM) while addressing security and privacy concerns. This approach contributes to the extended definition of TAM in wearable technology, offering new pathways for bio-mimetic research in smart devices and robotics.	Page 1
**INTRODUCTION**			
Rationale	3	Despite the increasing interest in wearable technology, the current literature has examined either the technical functions of DWPs [16] or the variables influencing customer acceptance separately [5]. This fragmented approach limited our understanding of how technological innovation and user perceptions interact. Furthermore, the exist-ing studies focused on the traditional technology acceptance model (TAM) and lacked emergent technical functions. For example, biomimetic principles, particularly biomimetic technological innovation, are underexplored in the context of DWP adoption models. With advancements in bio-inspired sensors, energy-efficient materials, bio-inspired structures, and adaptive interfaces, integrating biomimicry principles into DWP design could enhance the understanding of TAM and enhance long-term sustainability while improving functionality and user experience [12]. Thus, there is a lack of a comprehensive understanding of the functions of wearable technology in the consumer acceptance model.	Page 3
Objectives	4	Therefore, this study made significant contributions to this gap by understanding the comprehensive technical functions of digital wearable products (DWPs) in the consumer acceptance model. A comprehensive systematic literature review with a bibliometric, descriptive, and thematic analysis was conducted to identify the types of DWPs in the consumer acceptance model; to understand the role of technical functions of DWPs in the consumer acceptance; to detect the related outcomes (contributions) of the DWPs in the consumer acceptance model.	Page 3

Section and Topic	Item #	Checklist item	Location where item is reported (Page No)
**METHODS**			
Eligibility criteria	5	(a) Including only studies published between 2014 and 2024 in the English language. Studies published before 2014 or in a language other than English were excluded. Based on the existing literature, the field of smart and digital wearable technology (such as smartwatches and fitness trackers) experienced significant growth and innovation in the mid-2010s (around 2014) [26,46].<br>(b) Study type: including only empirical research articles (quantitative, qualitative, or mixed-methods) published in peer-reviewed H-index journals. Review articles, conference papers and proceedings, books, reports, or other text materials not published in peer-reviewed, indexed journals were excluded to ensure the quality and reliability of the selected studies [40,41].<br>(c) Study scope: selected studies must include results on the technical function (factors) of DWPs and the context of consumer acceptance and/or human perceptions and outcomes. Studies that did not address at least one result on the technical functions of DWPs were excluded to ensure relevance to the research questions.<br>(d) Target group: The sample of the studies must include adult individuals aged 18 years and over. This age range is targeted due to their higher propensity to adopt new technologies and their significant representation in the consumer market for digital wearable products [26,46]. The inclusion and exclusion criteria were applied in different screening and identification phases of this systematic review using the Mendeley Desktop, version 1.19.8.	Page 10
Information sources	6	Three electronic databases were reviewed:<br>(1) WoS<br>(2) Scopus<br>(3) ScienceDirect<br>A manual search was conducted for the references of the complete full-text content using Google Scholar.	Page 8
Search strategy	7	Three key variables were used to generate the keywords from the databases, including the exposure (digital wearable products [keywords] and their technical functions[keywords], outcome (consumer acceptance [keywords], and control or moderator (consumer behavior [keywords]). Therefore, the following search string and keywords were used in the current systematic review: ("digital wearable products" OR "smart wearable technology" OR "wearable devices" OR wearables OR "wearable technology" OR "smartwatches" OR "fitness trackers" OR "trackers" OR "smart glasses" OR "clothing" OR "rings" OR "jewelry" OR sensor) AND/OR ("technical functions" OR "functional features" OR "technical capabilities" OR "perceived usefulness" OR "ease of use" OR factors OR functions) AND (intention OR acceptance OR adoption OR perception Or behavior* OR behaviour* OR attitude).<br>Truncation, Boolean operators, parentheses, wildcards, quotation marks, and MeSH terms or search terms related to the described keywords were applied whenever possible.	Page 8
Selection process	8	In the first stage of PRISMA (identification), two reviewers (Author 1 and Author 2) independently searched the selected databases using the Electronic Management Research Library Database of the two reviewers' affiliated universities. They imported the retrieved records from all fields into Mendeley Desktop. The search was limited to English-language articles published between 2014 and 2024 that focused on the technical functions of DWPs. Duplicate records were identified and removed at this stage. In the second stage	Page10

Section and Topic	Item #	Checklist item	Location where item is reported (Page No)
		(screening), the two reviewers screened the imported records based on the type, year of publication, and language. Subsequently, the titles and abstracts were further screened based on the study scope criteria. Studies were included if they ad-dressed the technical functions of DWPs in the context of consumer acceptance and/or human perception and outcomes. Articles selected by at least one reviewer were retained for further assessment. In the third stage (eligibility), the selected articles were retrieved in full text and evaluated based on their relevance to the scope, type of participants, methodology, and quality appraisal of the study. A manual reference search was also conducted using Google Scholar to identify any additional relevant studies. At the last step, the two independent reviewers thoroughly debated the selected articles and agreed on their inclusion; any disagreements regarding study inclusion were re-solved through discussion with the third reviewer (Author 3). A summary table was created for the selected full-text articles to present the data from each study. The data analysis and synthesis were conducted by the first and second reviewers (Author 1 and Author 2) using At-las.ti.9 and VOSviewer 1.6.20 software. All authors approved the procedures followed in the search protocol.	
Data collection process	9	The first step of the data extraction and analysis was conducting a descriptive bibliometric analysis on the key information of the selected studies (such as year, source, methodology, and country of the selected studies). Besides, a summary table was generated to show the main information of the selected studies (such as title and objective of the study, country and source of the study, wearable type, methodology, and results, see Appendix A, Table A1). In the second step of the analysis, a bibliometric analysis was also conducted on the full-text selected papers to identify the key knowledge that helped to identify the specific themes, categories, and codes related to the review topic, in turn helping in conducting valid thematic and descriptive analysis and synthesis for the systematic review.<br><br>The bibliometric analysis was conducted on 38 full-text selected papers using co-authorship and keyword co-occurrence network for the author and keyword links analysis (using total strength links, occurrences, and frequency). This analytical approach is valuable as it identifies common viewpoints among the publications and authors, therefore guiding in determining the topic themes, related terms, and knowledge structures. For the graphical mapping of keyword co-occurrence networks, network theory was employed, with clustering methods determined by Waltman et al. [42]. All calculations were performed using Microsoft Excel and VOSviewer version 1.6.20[43].<br><br>The last step of the analysis was conducting a descriptive thematic analysis (extracting and synthesizing the data based on the theme, category, and codes, and de-scribing the extracted data using frequency, tabular, and figurative analysis) on the key themes, categories, and codes identified from the previous steps [41].	Page 10-11
Data items	10	Based on the theoretical model of the review, bibliometric analysis results, and in line with the research questions, five main themes were identified for data extraction and data analysis: (a) digital wearable products (DWPs), (b) technical functions of DWPs, (c) consumer acceptance, (d) user behavior in the consumer acceptance model, and (d) perceived outcomes of the consumer acceptance model. The descriptive analysis of the first theme helped to answer the first question of this systematic review; the second, third, and fourth themes aided in answering the second question. Meanwhile, the last theme guided in answering the third research question.<br><br>The first theme (DWPs) was described in 8 codes related to the types of DWPs and their connections to the categories and codes of technical functions (second theme), consumer acceptance (third theme), and user	Page 11

Section and Topic	Item #	Checklist item	Location where item is reported (Page No)
		behavior (fourth theme). The second theme (technical functions of DWPs) was described in four key categories and 20 codes. The four categories were wearable technology (including PU and PEOU), appearance and design, biomimetic innovation, and security and privacy. The third theme (consumer acceptance) was used to describe the concept of consumer acceptance in the DWPs context and their related factors in line with the second and fourth themes. The fourth theme (user behavior in the consumer acceptance model) was discussed in four key codes, including personal factor, social factor, attitude of use, and behavioral intention. It also addressed the relationship between the user behavior codes and the technical functions of DWPs in the consumer acceptance mode. The fifth theme (perceived outcomes of the consumer acceptance model) was described in five key categories and eight codes; the five categories included health and fitness, enjoyment, social value, biomimicry application, and market growth.	
Study risk of bias assessment	11	Two reviewers independently carried out a quality assessment of the included studies using the Newcastle-Ottawa Scale (NOS). Any disagreements regarding the included studies were resolved in an online meeting among the authors. The NOS is a risk-of-bias tool for nonrandomized studies, which assesses three domains, including study selection (four factors), study comparability (one factor), and exposure and outcomes (two factors). In general, studies with a low risk of bias (NOS score from 6 to 7 points) were included in the current review. Studies with a high risk of bias (NOS score from 4 to 5 points) and a very high risk of bias (NOS score from 0 to 3 points) were excluded.	Page 12
Synthesis methods	13a	All studies were reviewed based on the mentioned five themes using thematic, descriptive, tabular, and figural analysis via Atlas.ti.9, Mendeley Desktop, and Microsoft Excel.	Page 10
	13b	All studies were analyzed based on the mentioned five themes using thematic, descriptive, tabular, and figural analysis via Atlas.ti.9, Mendeley Desktop, and Microsoft Excel.	
	13c	Atlas.ti.9, VOSviewer 1.6.20, and Microsoft Excel were used for the statistical data in each theme.	
	13d	Each study result was analyzed individually to extract the relevant data.	
Reporting bias assessment	14	Specify any assessment of risk of bias that may affect the cumulative evidence.	Page 12
Certainty assessment	15	Two reviewers independently carried out a quality assessment of the included studies using the Newcastle-Ottawa Scale (NOS), as mentioned above.	
**RESULTS**			
Study selection	16a	A total of 1,209 records were identified through the search utilizing the chosen databases. A manual search of the reference lists of the selected full-text papers and Google Scholar added seven additional articles. After removing duplicate studies and including only peer-reviewed journal articles published in the English language from 2014 to 2024, a total of (n=942) papers were retained. However, (n=448) publications were rejected after title screening, and (n=293) were declined following abstract screening, as they were considered ineligible due to the unsuitable scope of the studies (n=622) or the unsuitable sample of the study (n=119). In the eligibility phase, the two reviewers meticulously examined a total of (n=201) full-text papers. Of them, (n=38) full-text articles were included in the thematic and descriptive analysis, while (n=163) articles were excluded. The ground for exclusion were as follows: (n=56) articles were review studies, (n=18) articles involved samples that	Results; Page 8; Lines 244-257.

Section and Topic	Item #	Checklist item	Location where item is reported (Page No)
		were not aged 18 years and above, (n=83) articles did not include any results on DWPs and consumer acceptance or human interaction, and (n=5) studies did not meet the required quality standards (NOS total quality score less than 6).	
	16b	Only five studies excluded due to failed in quality appraisal (NOS total quality score was less than 6). The rejected studies mostly could not provide information on outcomes, ascertainment of exposure, controlled confounding factors, or sample size.	Results; Page 8; Lines 244-257.
Study characteristics	17	For the individual studies, the frequency of the publication year showed that most of the selected papers were published in 2023 (n=9, 23.68%) studies, followed by 2020 (n=6, 15.78%) studies, and 2021 and 2023 (n=5, 13.15%) studies each. The data reveals a gradual increase in studies on this topic beginning around 2017.<br>Regarding the studies' sources, the 38 publications included in the review were published in (n=31) sources. The top five sources with the highest number of publications were: Technology in Society (n=3 references), Computers in Human Behavior (n=3 references), Digital Health (n=2 references), International Journal of Human–Computer Interaction (n=2 references), and npj Digital Medicine (n=2 references), see Table 1. Table 1 also shows that the pioneer publisher in the field was Sensors from MDPI, with a publication in early 2014; meanwhile, the most recent publication was published in Frontiers in Public Health.<br>Most of the selected studies (n=29, 76.31%) had adopted quantitative methods, applying a variety of data collection methods, including questionnaire survey (n=17, 44.73%), experimental approaches (n=9, 23.68%), observation study (n=2, 5.26%), and mixed quantitative survey and experimental approaches (n=1, 2.63%) study, see Figure 3 and Appendix A, Table A1. Followed by seven qualitative studies (n=7; 18.42%) applied four main qualitative methods, including case study and interviews (n=3, 7.89%), diary interviews (n=2, 5.26%), netnography (n=1, 2.63%), and in-depth expert interviews (n=1, 2.63%) study. Two studies were mixed methods (n=2; 5.26%) using a systematic review and questionnaire survey (n=1, 2.63%) and online survey and focus group discussions (n=1, 2.63%) study. Research strategy is critical in outlining the general approach re-searchers take to address their research questions.<br>Overall, the selected studies were conducted in (n=13) countries around the world. Most of the selected studies have been done in the USA (n=11, 28.94%) studies, followed by China (n=9, 23.68%) studies, South Korea (n=4, 10.52%) studies, Australia and Malaysia (n=2, 5.26%) studies each, Mexico, Brazil, India, Bangladesh, Turkey, Italy, Ger-many, and South Africa (n=1, 2.63%) study each. Besides, one of these studies was conducted in a global context, including Europe, America, Asia, and Australia (n=1, 2.63%) study.	Page 12-14
Risk of bias in studies	18	All selected study applied certain level of validity, Supplementary information S1.	Supplementary information S1
Results of individual studies	19	Individual studies data is presented in Supplementary information S1.	Supplementary information S1.
Results of syntheses	20a	(1) DWPs<br>Regarding the research question one (RQ1), it identified the types of DWPs and the related technical function of each type in the context of consumer acceptance. This systematic review revealed eight significant types of DWPs in the digital consumption market, including smartwatch, medical devices and robotics, wearable fitness	Page 26-31

Section and Topic	Item #	Checklist item	Location where item is reported (Page No)
		devices, wearable fashions, smart glass, wristwatch accelerometer, sports wearables, and other general wearable technology. These different types of DWPs played a pivotal role in shaping the model of consumers' perceptions, acceptance, and behavioral engagement in the digital products market [14, 18].<br>(2) Technical function of DWPs<br>Concerning research question two (RQ2), the thematic analysis revealed that the technical functions of the DWPs can be divided into four functions: (1) wearable technology factors (perceived usefulness and ease of use), (2) appearance and design, (3) biomimetic innovation, and (4) security and privacy concerns, see Figure 8. These findings align with and extend existing technology adoption theories, such as the Technology Acceptance Model (TAM), while highlighting unique considerations for wearable devices [37]. Regarding the first function, wearable technology, mirrors the core constructs of TAM and includes two sub-functions: (a) perceived usefulness (PU), which includes health monitoring, fitness tracking, lifestyle monitoring, data feedback, artificial intelligence (AI), and productivity; and (b) ease of use (PEOU), including ease of use and wearing comfort. The review findings demonstrate that both PU and PEOU served as significant predictors of consumer acceptance for wearable technologies and digital robotics. The AI, an emerging discipline, also played an increasingly vital role as a technological function in wearable items and robots, considerably impacting customer acceptance to embrace and continue using these technologies. It expanded the device intelligence by providing context-aware services, real-time analytics, predictive monitoring, and personalized feedback, which increased the perceived usefulness of these devices[57,76]. These results were in line with Chun et al. [48], who identified key technological aspects for the smartwatches, revealing the importance of health and fitness tracking functions, data synchronization, and AI in promoting consumer intentions towards these DWPs. The findings also contribute to Ma et al. [30] findings, which revealed that comfort and seamless integration with everyday routines were critical for the long-term usage of multifunctional wearable sensors, indicating that ease of use in wearables must be reconceptualized to include ergonomic elements..<br>(3) Related outcomes to consumer acceptance model<br>In addressing research question three (RQ3), this study identified five significant types of perceived outcomes (contributions) of the DWPs in the acceptance model, including (a) health and fitness, (b) enjoyment outcome, (c) social value, (d) biomimicry application, and (e) market growth. The most commonly reported perceived advantage was health and fitness outcomes, referring to the DWPs' abilities in enhancing and monitoring users' health and fitness, aligning with the primary (five) functionalities of the digital wearable products, including smartwatches, fitness trackers, medical devices, and robotics. Previous research confirms that health monitoring features, including heart rate, sleep, and physical activity tracking, enhance user motivation and intention for sustained usage, thus contributing to general health. Muzny et al. [21] and Lu et al. [25] highlighted that wearable health technologies facilitate behavioral modification and preventative healthcare, rendering them especially attractive to health-conscious consumers.	
Reporting biases	21	All selected study applied certain level of validity, Supplementary information S1.	Supplementary information S1
Certainty of evidence	22	High, refer to the inclusion/exclusion criteria above.	NA

Section and Topic	Item #	Checklist item	Location where item is reported (Page No)
**DISCUSSION**			
Discussion	23a	In line with the research questions, the objective of this systematic review was to identify the types of DWPs in the consumer acceptance model; to understand the role of technical functions of DWPs in the consumer acceptance; to detect the related outcomes (contributions) of the DWPs in the consumer acceptance model. To answer the research questions, a comprehensive systematic literature review with a bibliometric, descriptive, and thematic analysis was conducted on the full-text of 38 indexed peer-reviewed journal articles published between 2014 and 2024. The bibliometric analysis was conducted first to provide a general understanding of the topic of WDPs in consumer acceptance. It helped to identify the specific themes and topics covered in the included studies. Therefore, the bibliometric analysis led to identify five theme that will be studied in the thematic analysis, including (a) digital wearable products (DWPs), (b) technical functions of DWPs as discussed, (c) consumer acceptance, (d) user behavior, and (e) perceived outcomes of the consumer acceptance model. However, the biblio-metric visualization analysis only focused on keywords and co-authorship analysis, which could not give the whole picture of the knowledge on the topic. Therefore, the further thematic analysis of the systematic review aimed to provide a better under-standing of the comprehensive multidimensional perspective of the DWPs in the consumer acceptance model.	Page 26-27
	23b	Regarding the research question one (RQ1), it identified the types of DWPs and the related technical function of each type in the context of consumer acceptance. This systematic review revealed eight significant types of DWPs in the digital consumption market, including smartwatch, medical devices and robotics, wearable fitness devices, wearable fashions, smart glass, wristwatch accelerometer, sports wearables, and other general wearable technology. These different types of DWPs played a pivotal role in shaping the model of consumers' perceptions, acceptance, and behavioral engagement in the digital products market [14, 18].	Page 26-27
	23c	Concerning research question two (RQ2), the thematic analysis revealed that the technical functions of the DWPs can be divided into four functions: (1) wearable technology factors (perceived usefulness and ease of use), (2) appearance and design, (3) biomimetic innovation, and (4) security and privacy concerns, see Figure 8. These findings align with and extend existing technology adoption theories, such as the Technology Acceptance Model (TAM), while highlighting unique considerations for wearable devices [37]. Regarding the first function, wearable technology, mirrors the core constructs of TAM and includes two sub-functions: (a) perceived usefulness (PU), which includes health monitoring, fitness tracking, lifestyle monitoring, data feedback, artificial intelligence (AI), and productivity; and (b) ease of use (PEOU), including ease of use and wearing comfort. The review findings demonstrate that both PU and PEOU served as significant predictors of consumer acceptance for wearable technologies and digital robotics. The AI, an emerging discipline, also played an increasingly vital role as a technological function in wearable items and robots, considerably impacting customer acceptance to embrace and continue using these technologies. It expanded the device intelligence by providing context-aware services, real-time analytics, predictive monitoring, and personalized feedback, which increased the perceived usefulness of these devices[57,76]. These results were in line with Chun et al. [48], who identified key technological aspects for the smartwatches, revealing the importance of health and fitness tracking functions, data synchronization, and AI in promoting consumer intentions towards these DWPs. The findings also contribute to Ma et al. [30] findings, which revealed that comfort and seamless integration with everyday routines were critical for the long-term usage of multifunctional wearable sensors, indicating that ease of use in wearables must be reconceptualized to include ergonomic elements.	Page 28-29

Section and Topic	Item #	Checklist item	Location where item is reported (Page No)
		In addressing research question three (RQ3), this study identified five significant types of perceived outcomes (contributions) of the DWPs in the acceptance model, including (a) health and fitness, (b) enjoyment outcome, (c) social value, (d) biomimicry application, and (e) market growth. The most commonly reported perceived advantage was health and fitness outcomes, referring to the DWPs' abilities in enhancing and monitoring users' health and fitness, aligning with the primary (five) functionalities of the digital wearable products, including smartwatches, fitness trackers, medical devices, and robotics. Previous research confirms that health monitoring features, including heart rate, sleep, and physical activity tracking, enhance user motivation and intention for sustained usage, thus contributing to general health. Muzny et al. [21] and Lu et al. [25] highlighted that wearable health technologies facilitate behavioral modification and preventative healthcare, rendering them especially attractive to health-conscious consumers.	Page 30-31
**OTHER INFORMATION**			
Registration and protocol	24	PROSPERO was searched to ensure a similar systematic review study protocol was not registered. No prior studies focusing on the current topic of interest were identified.	-
Support	25	This research was funded by the “GP-IPM/2023/9746100” number (9746100) from RMC, Universiti Putra Malaysia, and “GERAN SWASTA/ANTARABANGSA” number (6300507).	Page 33
Competing interests	26	The authors declare that they have no competing interests.	Page 33
Availability of data, code and other materials	27	Supplementary documents have been attached to the manuscript.	Supplementary information 1, 2, & 4.

From: Page MJ, McKenzie JE, Bossuyt PM, Boutron I, Hoffmann TC, Mulrow CD, et al. The PRISMA 2020 statement: an updated guideline for reporting systematic reviews. BMJ 2021;372:n71. doi: 10.1136/bmj.n71
