# Supplementary material for: Technical Functions of Digital Wearable Products (DWPs) in the Consumer Acceptance Model: A Systematic Review and Bibliometric Analysis with a Biomimetic Perspective
Source: Biomimetics (Basel). 2025 Jul 22;10(8):483. doi: 10.3390/biomimetics10080483 (PMC12383983; doi:10.3390/biomimetics10080483)
Supplement: Supplementary file 1 [file biomimetics-10-00483-s001.zip › biomimetics-3722362-supplementary/Supplementary Material_S2.pdf]

## Supplementary Document 1

### Summary data extraction and synthesis

The following Table were developed in Microsoft Excel 365, to show the key extracted data of this systematic review, including Study ID, Author(s), Source Type, Publication Year, Country, Title of the Study, Objective, Methodology, Variables/ Themes, Theory/Framework, Wearables Type, Trends and Limitations. These items were considered based on the defined research questions and the goals of this study. The description of these items is presented in Table 1.

**Process data extracted for the full-text selected papers.**

| Data | Description |
|---|---|
| ID | Unique identifier for each primary study. |
| Author(s) | Names of the study's authors. |
| Source Type | Type of source (journal article) |
| Publication Year | The year of publishing the paper. |
| Country | Country where the study was conducted. |
| Title | Study's title. |
| Objective | Main objective of the study. |
| Methodology | Design science, quantitative, qualitative, mixed method etc. |
| Variables/ Themes | Key factors or concepts analyzed in the study. |
| Theory/Framework | The theory or framework that the study was adopted such as TAM, DOI, UTAUT etc. |
| Results | Key information and data related to the study themes, categories and codes. |
| Wearables Type | The type of wearables that were explored in the primary studies such as fitness trackers, smart watches, smart jewelry, etc. |
| Trends | Evolving directions in the field. |
| Limitations | Constraints identified in the study. |

**Supplementary Document 1**

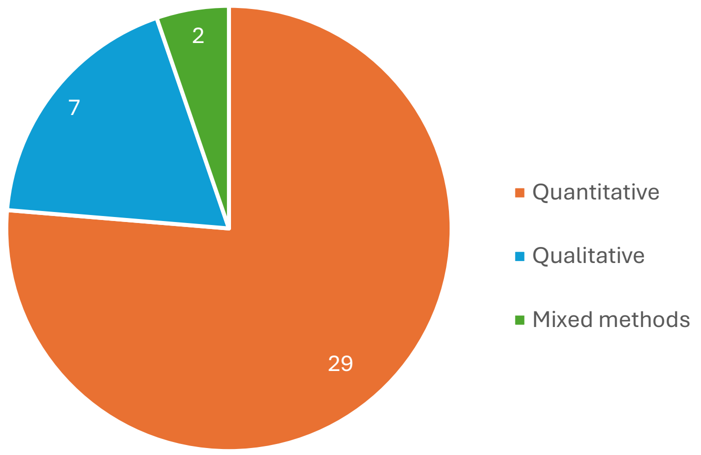


**Figure.** Distribution of research methodologies, based on frequency (number of studies)
