# Supplementary material for: Technical Functions of Digital Wearable Products (DWPs) in the Consumer Acceptance Model: A Systematic Review and Bibliometric Analysis with a Biomimetic Perspective
Source: Biomimetics (Basel). 2025 Jul 22;10(8):483. doi: 10.3390/biomimetics10080483 (PMC12383983; doi:10.3390/biomimetics10080483)
Supplement: Supplementary file 1 [file biomimetics-10-00483-s001.zip › biomimetics-3722362-supplementary/Supplementary Material_S4.pdf]

Supplementary Document 4

Table S4. Quality assessment using the Newcastle-Ottawa Scale (NOS).

Study	Selection				Comparability	Outcome		Score	Accepted
	Representativeness of the Sample	Sample Size Justified	On-Respondents	Ascertainment of Exposure (Max**)	Confounding Controlled (Max**)	Assessment (Max**)	Statistics	Total	
Study 1	*			**	*	**	*	7	✓
Study 2	*			**	*	**	*	7	✓
Study 3	*	*		**	*	**	*	8	✓
Study 4	*	*		*		*	*	5	✗
Study 5	*	*		*	*	**	*	7	✓
Study 6	*	*	*	**	**	**	*	10	✓
Study 7	*	*		**	*	**	*	8	✓
Study 8	*		*	*		*	*	5	✗
Study 9	*	*			*	*	*	5	✗
Study 10	*			**	*	**	*	7	✓
Study 11	*	*		*	*	**	*	7	✓
Study 12	*	*		**	*	**	*	8	✓
Study 13	*			**	*	**	*	7	✓
Study 14	*			*	*	*	*	5	✗
Study 15	*	*		*	*	**	*	7	✓
Study 16	*	*		*	*	**	*	7	✓
Study 17	*	*		*	*	*	*	6	✓
Study 18	*	*		*	*	**	*	7	✓
Study 19	*	*	*	**	*	**	*	10	✓
Study 20	*	*		*	*	**	*	7	✓
Study 21	*	*		*	*	**	*	7	✓
Study 22	*	*		*	*	**	*	7	✓

Study 23	*			**	*	**	*	7	✓
Study 24	*	*		*	*	**	*	7	✓
Study 25	*	*		*	*	**	*	7	✓
Study 26	*	*		*	*	**	*	7	✓
Study 27	*	*		*	*	**	*	7	✓
Study 28	*	*		*	*	**	*	7	✓
Study 29	*	*		*	*	**	*	7	✓
Study 30	*	*		*	*	**	*	7	✓
Study 31	*	*		*	*	**	*	7	✓
Study 32	*	*		*	*	**	*	7	✓
Study 33	*	*	*	*	**	**	*	9	✓
Study 34	*	*		*	*	*	*	5	✗
Study 35	*			*	*	**	*	7	✓
Study 36	*			**	*	**	*	7	✓
Study 37	*	*		*	*	**	*	7	✓
Study 38	*	*		*	**	*	*	7	✓
Study 39	*	*		*	*		*	4	✗
Study 40	*	*		*	**	**	*	8	✓
Study 41	*	*		*	**	**	*	8	✓
Study 42	*	*		*	*	**	*	7	✓
Study 43	*	*		*	*	**	*	7	✓

High quality = 6–10; moderate quality = 4–5; low quality = 0–3. The study should score at least “6” to be included in the review. * = moderately addressed; ** = strongly addressed; ✓ = accepted; ✗ = rejected.
